# Assembly and dynamics of the apple carposphere microbiome during fruit development and storage

**DOI:** 10.3389/fmicb.2022.928888

**Published:** 2022-08-09

**Authors:** V. Yeka Zhimo, Ajay Kumar, Antonio Biasi, Ahmed Abdelfattah, Vijay Kumar Sharma, Shoshana Salim, Oleg Feygenberg, Rotem Bartuv, Shiri Freilich, Susan R. Whitehead, Michael Wisniewski, Samir Droby

**Affiliations:** ^1^Department of Postharvest Science of Fresh Produce, Agricultural Research Organization, The Volcani Center, Rishon LeZion, Israel; ^2^Leibniz Institute for Agricultural Engineering and Bioeconomy (ATB), Max-Eyth Allee, Potsdam, Germany; ^3^Department of Natural Resources, Institute of Plant Sciences, Agricultural Research Organization, Newe Yaar Research Center, Ramat Yishay, Israel; ^4^Faculty of Agriculture, The Robert H. Smith Institute of Plant Sciences and Genetics in Agriculture, The Hebrew University of Jerusalem, Rehovot, Israel; ^5^Department of Biological Sciences, Virginia Polytechnic Institute and State University, Blacksburg, VA, United States

**Keywords:** apple, assembly, dynamics, microbiome, succession

## Abstract

Microbial communities associated with fruit can contribute to quality and pathogen resistance, but little is known about their assembly and dynamics during fruit development and storage. Three apple cultivars growing under the same environmental conditions were utilized to examine the apple carposphere microbiome composition and structure at different developmental stages and storage. There was a significant effect (Adonis, *p* ≤ 0.001) of fruit genotype and its developmental stages and storage times on the fruit surface microbial assemblage and a strong temporal microbial community succession was detected (Mantel test: *R* ≤ 0.5, *p* = 0.001) in both bacterial and fungal communities. A set of 15 bacterial and 35 fungal core successional taxa and members exhibiting differential abundances at different fruit stages were identified. For the first time, we show the existence of underlying universal dynamics in the assembly of fruit-associated microbiomes. We also provide evidence of strong microbial cross-domain associations and uncover potential microbe-microbe correlations in the apple carposphere. Together our findings shed light on how the fruit carposphere assemble and change over time, and provide new insights into fruit microbial ecology.

## Introduction

Microbial communities in ecosystems comprise complex assemblages of taxa that can establish beneficial, neutral, or detrimental interactions among themselves and with their host in the ecological niches they occupy (Little et al., 2008; Vorholt, 2012; Nemergut et al., 2014; Ghoul and Mitri, 2016; Hassani et al., 2018). In plant systems, microbial communities are found on the surface or inside plant tissues and organs, and their interactions with their hosts have impacted their colonization, evolution, and diversity (Ivanov et al., 2012; van Schie and Takken, 2014; Skiada et al., 2020; Delaux and Schornack, 2021). The microbial habitat associated with fruit (carposphere), similar to other plant parts, harbors a wide diversity of bacteria, archaea, fungi, and viruses, and their composition and diversity continues to be explored (Droby and Wisniewski, 2018; Abdelfattah et al., 2020, 2021; Piombo et al., 2020; Kumar et al., 2021). In contrast to the rhizosphere (the soil-root interface), where fluctuations in environmental conditions are often dampened by the bulk soil, above-ground parts of plants, such as the carposphere, represent a unique ecological system where the environment is much more dynamic and unstable (Droby and Wisniewski, 2018). Resident microbes in commercial fruit crops are exposed to large fluxes in abiotic conditions and rapid changes in the resource environment during fruit development and after harvest when the fruit are placed in storage. The carposphere is also prone to additional perturbations during various cultural and plant protection management practices. In this regard, it is unclear if ecological processes and models reported for either the rhizosphere or phyllosphere can be extrapolated to conditions prevailing in the carposphere. Interactions between fruit and its resident microbiota may include antagonistic interactions that result in active or latent infections by pathogenic microbes, as well as commensalistic or mutualistic interactions. Questions about the role and function of these microbial interactions in fruit quality and disease resistance are just beginning to be investigated (Diskin et al., 2017; Kusstatscher et al., 2020; Sare et al., 2021; Zhang et al., 2021; Zhimo et al., 2021; Sangiorgio et al., 2022). However, we still lack fundamental knowledge about the assembly, ecology, and community dynamics of fruit-associated microbiota in horticultural fruit crops, during different phenological and physiological stages or during storage. The acquisition of such information could inform the development of new tools like identification of appropriate antagonist microbes and designing synthetic communities that can be specifically formulated to target specific pathogens as an intervention during pre-harvest or postharvest storage, thereby reducing decays and prolong shelf life of fruits.

Studying community succession (defined here as series of progressive changes in the composition of an ecological community over time) is a fundamental pursuit in microbial ecology research (Fierer et al., 2010; Faust and Raes, 2012; Brislawn et al., 2019; Pascual-García and Bell, 2020). While each microbial community is distinct and subject to their specific environments, some generalized principles exist that shape their assembly and succession (Nemergut et al., 2013; Pascual-García and Bell, 2020). The overall variation in community structure and diversity (i.e., beta-diversity) that occurs during microbial community succession reflects two divergent phenomena: turnover and nestedness (Baselga, 2010). Turnover is an ecological process through which existing taxa are replaced with new ones and may reflect species sorting by environmental shifts through space or time, leading to selective differentiation of taxa pools among assemblages (Baselga, 2010; Victorero et al., 2018; Brislawn et al., 2019; Wang et al., 2019). In contrast, nestedness accounts for taxa loss or gain without replacement, where one community is a subset of another community, and may originate from processes of ordered loss or colonization across temporal and spatial gradients (Victorero et al., 2018; Brislawn et al., 2019; Wang et al., 2019; Liu and Müller, 2020).

Another question to address in fruit-associated microbial communities is the concept of universality in microbiomes, initially reported to exist in humans (Bashan et al., 2016), and recently in some arbuscular mycorrhizal (AM) fungal communities, where the existence of universality is dependent on ecosystem types (Van Geel et al., 2017; Verbruggen et al., 2018). Universality describes the extent to which there are consistent underlying dynamics that govern microbial community assembly across different hosts, sites, and ecosystems. Do universal dynamics govern fruit associated microbiomes in different ecosystems (geographic locations, fruit types, fruit developmental stages, management interventions) or does each community have its own unique individual set of dynamics? Universality can be identified by a signature in which the similarity in community composition across samples is linked to the similarity in abundance profiles of shared species (Bashan et al., 2016). This outcome suggests that there are regularities in community assembly across hosts, and that taxa within these communities interact in a similar manner. If this holds true for fruit-associated communities, it would suggest that a method of microbiome manipulation valid in one system is likely to work in other systems.

**Figure 1:**
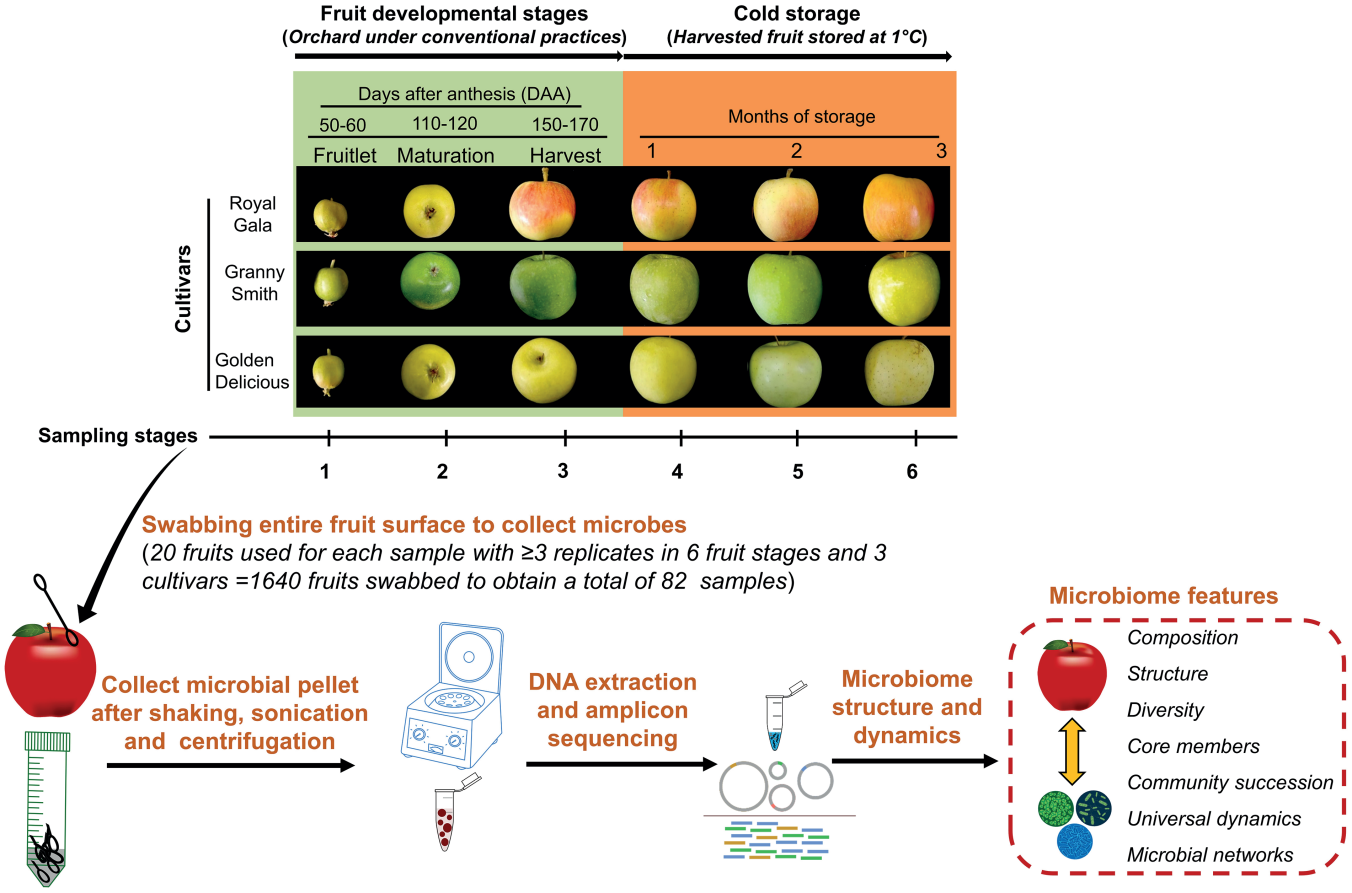
Schematic illustration of the experimental set-up. The apple fruit microbiomes were sampled from three cultivars namely “Royal Gala,” “Granny Smith” and “Golden Delicious” growing in the same orchard and plot where they received a conventional maintenance program. Sampling was carried out in the field during three developmental stages: fruitlet (50–60 days after anthesis; DAA), maturation (110–120 DAA) and at harvest (150–170 DAA) and another three times from the harvested fruit lot during cold-storage periods (1°C) at monthly intervals. Fruits were swabbed, microbial pellets were collected by washing the swabs and concentrated from 20 fruits each to represent a sample. Genomic DNA was extracted from these samples, sequenced and processed to obtain microbiome features.

The fruit surface of the domesticated apple (*Malus* × *domestica* Borkh.) has been reported to be naturally colonized by numerous microbes that vary in abundance and diversity according to host genotype, geographical location, management practices, and fruit tissue type (Wassermann et al., 2019a,b; Abdelfattah et al., 2020, 2021; Bösch et al., 2021). Little is known, however, about the ecological processes, particularly with regard to selection, that regulate temporal carposphere microbial dynamics. Therefore, we designed an experiment utilizing three commercial apple cultivars growing in the same geographical location and management program, and followed the epiphytic bacterial and fungal communities of the fruit throughout several developmental stages and for a period of time after harvest when the apples were in storage (Figure 1). The three cultivars used in the study “Royal Gala,” “Golden Delicious” and “Granny Smith” are among the most popular cultivars grown worldwide with significant economic importance. “Granny Smith” and “Golden Delicious” have different genetic lineages arising from chance seedlings while “Royal Gala” is a product of traditional breeding between “Kidd’s Orange Red” and “Golden Delicious.” We aimed to address the following objectives: (1) determine the role of host factors, such as genotype, fruit developmental stage and postharvest storage, in shaping the microbial assembly and dynamics of the apple carposphere, (2) explore patterns of community succession and quantify the relative contribution of turnover and nestedness components, and (3) elucidate the underlying ecological networks and microbial associations that govern carposphere microbial assembly and dynamics of the apple carposphere microbial assembly.

## Results

### Influence of genotype and dynamics of carposphere microbial community assemblage at different fruit developmental stages and storage period

**Figure 2:**
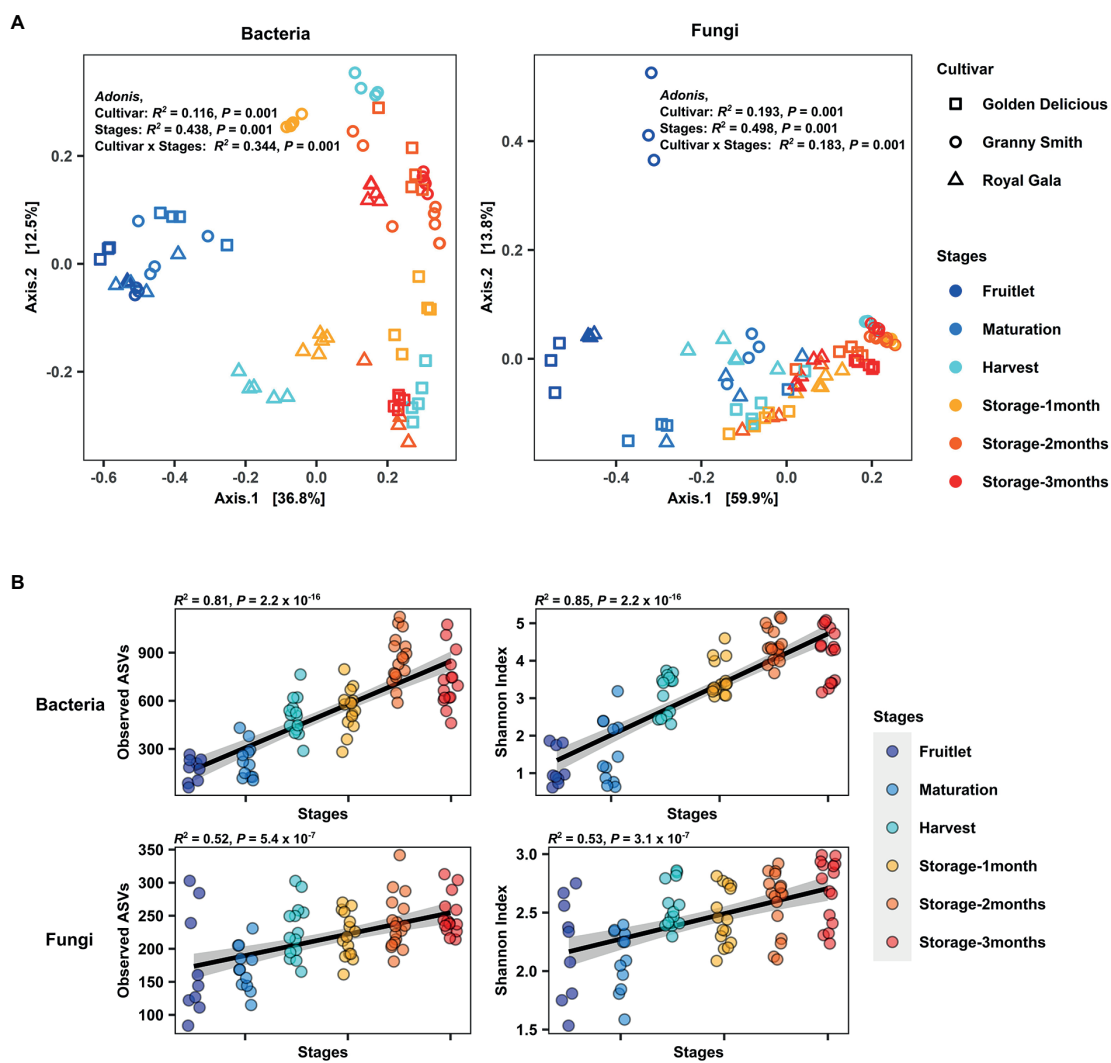
Overview of the apple carposphere microbiome. **(A)** Principal coordinate (PCoA) analysis of bacterial and fungal community based on Bray–Curtis dissimilarity with permutational analysis of variance (PERMANOVA) showed significant association of bacterial and fungal community compositions with fruit stages (developmental stages and storage period) and cultivar type (Adonis, *p* < 0.001). **(B)** Strong positive correlation (Spearman, *p* < 0.0001) between the fruit developmental stages and storage periods (plotted at the *x*-axis) and bacterial and fungal richness (Observed ASVs) as well as Shannon diversity (plotted at the *y*-axis) in the apple carposphere microbiome.

Differences in microbial composition among sample groups were assessed using permutational multivariate analysis of variance PERMANOVA and principal coordinate analysis (PCoA) based on Bray–Curtis dissimilarity. The *R*^2^ values of PERMANOVA (Adonis) were used to explain the variance components of the different factors. A PERMANOVA model with “Stages” representing the fruit stage, “Cultivar” representing the fruit genotype and their interaction “Cultivar × Stages” as explanatory factors was used to examine the factors shaping overall variation in microbial community composition. We found that overall, “Stages” explained most of the microbial community variation (Adonis, bacteria: *R*^2^ = 0.44, *p* = 0.001; fungi: *R*^2^ = 0.50 *p* = 0.001), while “Cultivar” explained a lesser degree of the variation (Adonis, bacteria: *R*^2^ = 0.12, *p* = 0.001; fungi: *R*^2^ = 0.19, *p* = 0.001). The interaction between the two factors (“Cultivar × Stages”) also exhibited significant impact on the variation between communities (Adonis, bacteria: *R*^2^ = 0.34, *p* = 0.001; fungi: *R*^2^ = 0.18, *p* = 0.001; Figure 2A).

The PCoA plot illustrates this effect with the samples largely separated by these two factors along principal coordinate axes 1 and 2 for both bacterial and fungal communities. When similar analyses were performed to check for differences in the community composition among stages separately for each cultivar (Supplementary Figure 3) or among cultivars separately for each stage (Supplementary Figure 4), significant differences (Adonis, *p* ≤ 0.01) in both bacterial and fungal communities in each of the groups analyzed were observed except for the fungal community at maturity stage among cultivars (*p* = 0.1689). Pairwise comparisons between cultivars during each stage revealed that in both bacterial and fungal communities, significant differences (Pairwise.adonis, *P* adjusted < 0.05) between cultivar pairs were observed only at the latter stage of fruit development (harvest) and during storage (Supplementary Table 3). Similar pairwise comparisons between individual stages revealed that the bacterial community differed in composition between most of the stage pairs (Pairwise.adonis, *P* adjusted < 0.05), although the *R*^2^ values explaining the variance components were lesser for storage period pairs as compared to pairs from early developmental stages (Supplementary Table 4). In the case of fungal community composition, significant differences were seen in pairwise comparisons between early developmental stage pairs (Pairwise.adonis, *P* adjusted < 0.05) except between maturation and harvest stages (Pairwise.adonis, *P* adjusted = 0.06), but no differences were seen for storage period pairs (Pairwise.adonis, *P* adjusted > 0.05). Conducting pairwise comparisons among each individual stages of each cultivar revealed significant differences between many stage pairs in the fungal community but little to no significant differences between stages in the bacterial community after corrections for multiple testing (Supplementary Table 5).

A multivariate homogeneity of group dispersions test (PERMDISP2) based on Bray–Curtis dissimilarity among the samples grouped by stage and cultivar was used to determine if there were differences in variance (beta-dispersion) between sample groups. Our results for bacterial communities revealed significant differences in variances among sample groups at different stages (Betadisper, bacteria: *p* = 0.003; fungi: *p* = 0.008), with lower variance (distance to centroid) at the early fruitlet stage compared to later stages of fruit development and during storage periods (Supplementary Figure 5A). Statistically significant differences in the bacterial community were only observed between the fruitlet and one-month of storage (Tukey HSD, *p* = 0.008) while in the fungal community, differences were seen between the fruitlet and 1-month of storage (Tukey HSD, *p* = 0.003), and between the fruitlet and 2-months of storage (Tukey HSD, *p* = 0.007). No significant differences in variances were observed between different cultivar groups (Betadisper, bacteria: *p* = 0.933; fungi: *p* = 0.491; Supplementary Figure 5B).

We then investigated α-diversity of the apple carposphere microbiome in samples by estimating within-sample richness (Observed amplicon sequence variants: ASVs) and Shannon diversity and using Spearman’s correlation to determine their association with fruit stages. We found a significant increase and strong positive correlation in richness and Shannon diversity with fruit stages in both the bacterial (richness: *R*^2^ = 0.81, *p* < 2.2 × 10^−16^; Shannon index: *R*^2^ = 0.85, *p* < 2.2 × 10^−16^) and fungal community (richness: *R*^2^ = 0.52, *p* = 5.4 × 10^−7^; Shannon index: *R*^2^ = 0.53, *p* = 5.3 × 10^−7^; Figure 2B). Interestingly, when analyses were conducted separately for the three cultivars, there were significant increases and positive correlations in both bacterial and fungal richness and Shannon diversity in all the three cultivars, except in “Royal Gala,” which did not show a positive correlation between fungal richness and diversity with fruit stage (Supplementary Figure 6). No significant differences were detected in overall α-diversity among the three cultivars (Kruskal–Wallis test) in the bacterial community (richness: *p* = 0.65; Shannon index: *p* < 0.56). Significant differences were observed, however, for fungal communities across cultivars in richness (*p* = 0.00018) but not in the Shannon Index (*p* = 0.073). Pairwise Wilcoxon tests among the three cultivars indicated that “Royal Gala” samples had a lower fungal richness and Shannon Index, relative to “Golden Delicious” and “Granny Smith” (Supplementary Figure 7). Collectively, these results confirm that the composition of microbial communities in the apple fruit is influenced by fruit genotype and display significant and dynamic assemblages at different fruit developmental stages and storage period.

### Community succession in apple carposphere microbial assemblages

**Figure 3:**
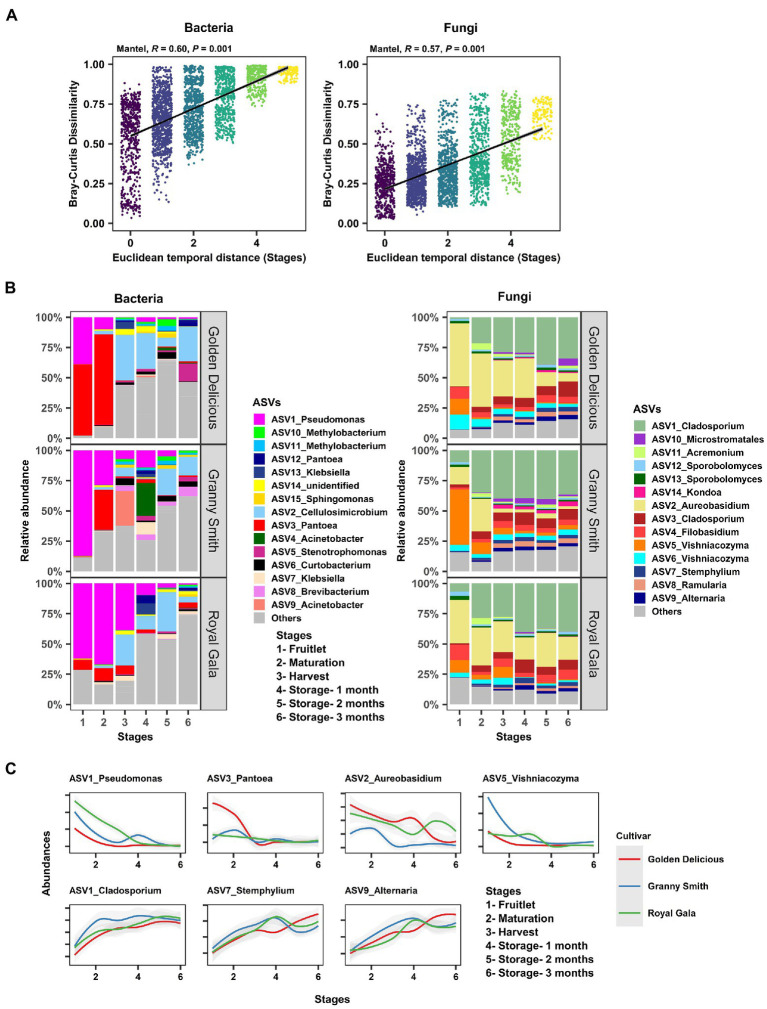
Community succession of the apple carposphere microbiome. **(A)** Mantel correlation between Euclidean temporal distance (fruit stages) and Bray–Curtis community dissimilarity showed strong succession in both bacterial and fungal communities. **(B)** Temporal change of the relative abundances of dominant bacterial and fungal amplicon sequence variants (ASVs) at each sampling stages in the carposphere microbiome of three apple cultivars as visualized using barplots. **(C)** Temporal change in the abundances of some ASVs corresponding to the genera *Pseudomonas*, *Pantoea*, *Aureobasidium*, and *Vishniacozyma* showed characteristic increasing trends in abundances while ASVs corresponding to genera *Cladosporium*, *Stemphylium*, and *Alternaria* showed decreasing trends in abundances in the carposphere of three apple cultivars.

After determining the effect of the host (fruit development stage, storage period, and genotype) on the microbial assemblage of the apple carposphere, we next investigated the dynamics of these microbial community assemblages over time. To do this, we performed a Mantel correlation analysis between temporal distances (based on Euclidean dissimilarity as periods between the six fruit sampling stages) and microbial community distances (based on Bray–Curtis community pairwise dissimilarity) from all sample pairs. Strong evidence of community succession was evident in both the bacterial (Mantel: *R^2^* = 0.60, *p* = 0.001) and fungal communities (Mantel: *R^2^* = 0.57, *p* = 0.001) in the carposphere microbiome over time (Figure 3A). Similar strengths of community succession was also seen when the sample pairs were analyzed separately for each of the three cultivars (Supplementary Figure 8). Confirmation of the results of these temporal dynamics in community composition was observed in the trends of relative abundance of the dominant bacterial and fungal ASVs (>1%) across fruit stages and cultivars (Figure 3B). Prominent declines in the relative abundance were observed of initially dominant ASVs belonging to the genera *Pseudomonas*, *Pantoea, Aureobasidium,* and *Vishniacozyma,* while increases in *Cladosporium, Stemphylium,* and *Alternaria* were evident, relative to the abundance of other genera (Figure 3C).

**Figure 4:**
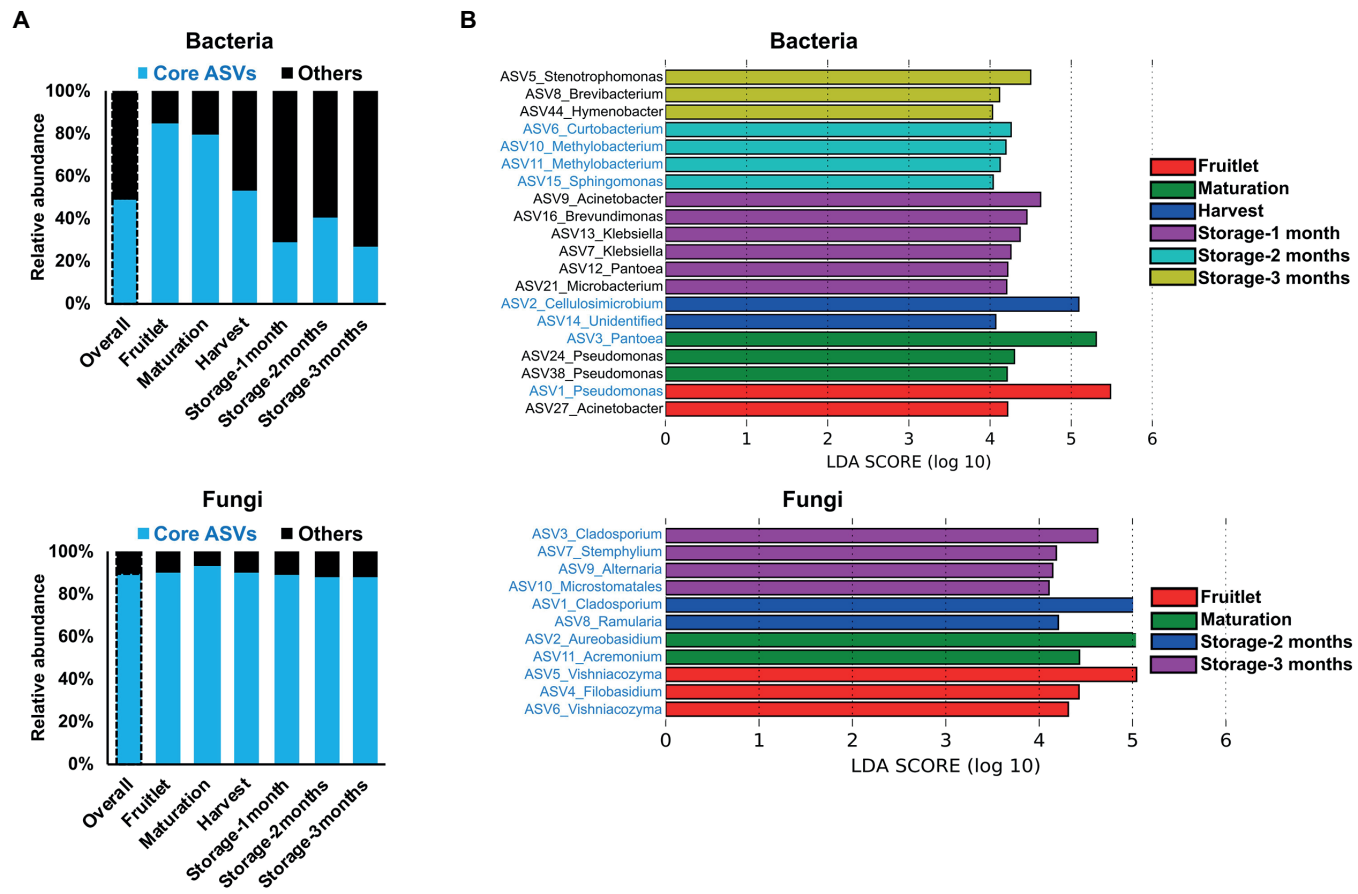
Core microbiome dynamics and distribution of the apple carposphere microbiome. **(A)** Distribution of core microbiome in the apple carposphere showed decreasing collective contribution of bacterial core amplicon sequence variants (ASVs) to the total bacterial abundance and a contrasting dominant and consistent contribution of fungal core ASVs to the total fungal abundance. **(B)** Identified biomarker ASVs (LDA score > 4.0, *p* ≤ 0.01) during different fruit stages using Linear Discriminant Analysis Effect Size (LEfSe) analysis comprised of both non-core and core ASVs in bacteria while all fungal biomarker ASVs comprised of only core ASVs. Biomarker ASV names in blue are core-members.

The dynamic nature of the apple carposphere microbiome structure over time raises questions regarding the role and fate of the core successional microbes and their persistence across different fruit stages. We identified 15 bacterial ASVs and 35 fungal ASVs, across all the sampling stages and cultivars, as core successional microbes based on 95% occupancy and no limit on the percentage of their contribution to relative abundance. Tracking these core microbes across the different fruit developmental stages and the storage period revealed contrasting dynamics in bacterial and fungal communities (Figure 4A). The collective contribution of the bacterial core microbes to the total abundance decreased dramatically over the course of the successive sampling times (from 84.2% during the fruitlet stage to 26.5% at the end of the storage period; Figure 4A). This trend was mainly due to dominance of two core members (ASV1_*Pseudomonas* and ASV3_*Pantoea*) during the early stages of fruit development followed by a dramatic decrease at the later fruit development stages, while another core member ASV2_*Cellulosimicrobium* became predominant at harvest and during the period of storage (Figure 3C). In contrast, the 35 ASVs that made up the fungal core microbiome consistently predominated in their collective abundance (contributing >85% of fungal abundance) throughout all of the fruit developmental stages and storage period (Figure 4A). Linear Discriminant Analysis Effect Size (LEfSe) analysis was used to determine taxa that most likely explained differences between the fruit stages based on their abundance. The analysis identified 20 bacterial and 11 fungal ASVs at different fruit stages that were differentially abundant (LDA score > 4.0, *p* ≤ 0.01; Figure 4B). Notably, all of the 11 fungal and some of the bacterial (8 out of 20) ASVs that differentiated the fruit stages were core taxa members. These results indicate that the core fungal and bacterial microbiomes are highly persistent and remain stable (especially in the fungal community) during community succession, potentially due to higher abundance of these microbes in the environment, leading to higher immigration and adaptation to the apple carposphere than the other members of the microbiome.

### Turnover drives community succession in the carposphere microbiome of apple

**Figure 5:**
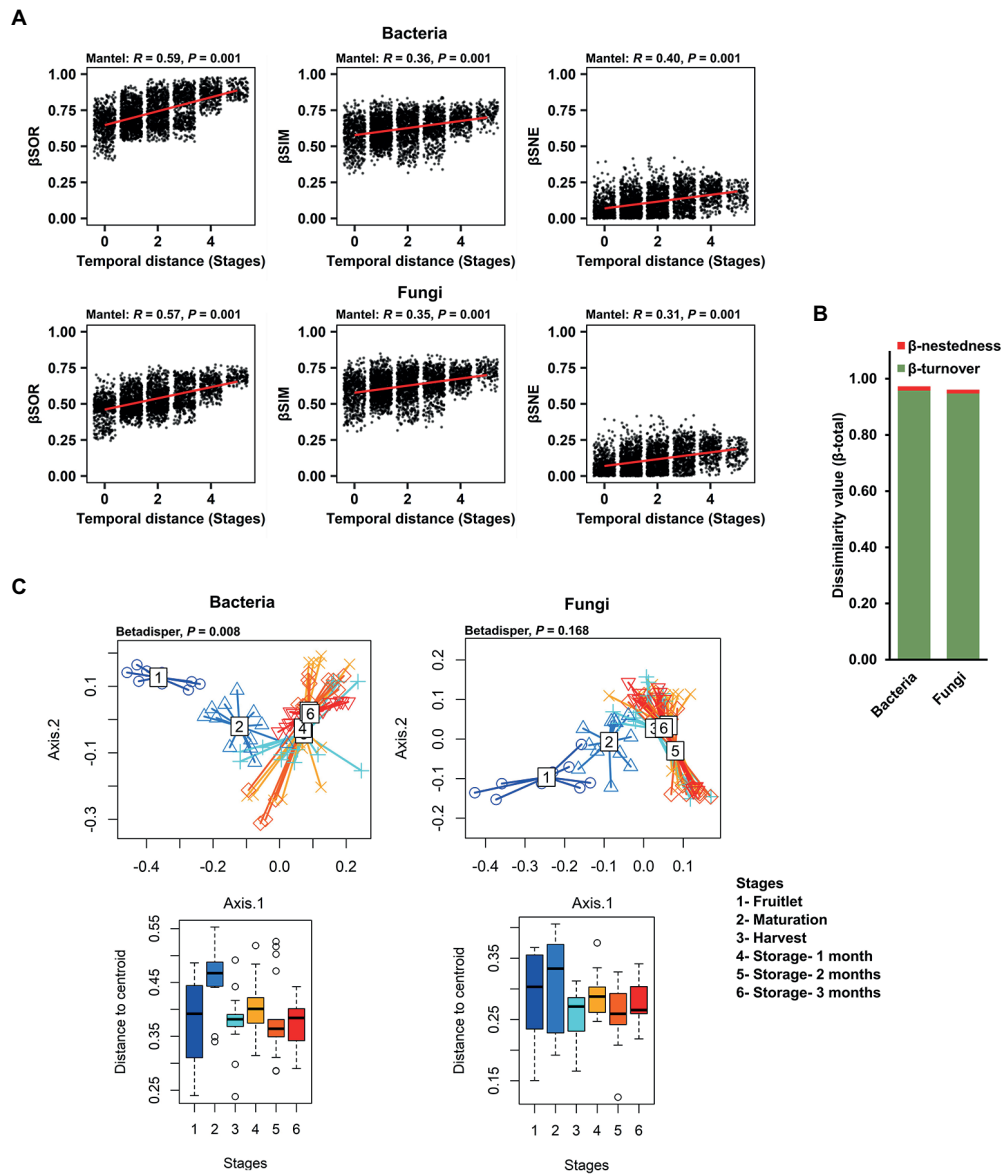
Role of two patterns, turnover and nestedness in the change in carposphere microbiome community composition over time. **(A)** Mantel tests to explore correlation of temporal distance (stages) with the compositional variance of bacterial and fungal community showed significant and biologically meaningful associations with the overall compositional variance (measured by Sorenson pairwise dissimilarity; β_SOR_) and also with both the turnover (Simpson pairwise dissimilarity; β_SIM_) and nestedness (Sorenson pairwise dissimilarity minus Simpson pairwise dissimilarity; β_SNE_) components after partitioning. The Sorenson and Simpson metrics differ from the Bray–Curtis metric in that they do not take into account relative abundances. **(B)** The overall compositional variance (β_-total_) was almost entirely contributed by the turnover component (β_-turnover_) rather than nestedness (β_-nestedness_) in the apple carposphere microbiome. **(C)** Turnover of both bacterial and fungal community composition through stages, demonstrated by PCoA plots using Simpson dissimilarity showed strong bacterial as well as fungal compositional turnover among stages 1–3 (developmental stages) followed by significantly less or no turnover for stages 4–6 (storage periods) but none were significantly different from other stages in case of the fungal community.

With the finding of strong community succession, despite the dominance and persistence of a few core ASVs, we explored the ecological processes underlying the strong community changes that impacted the rest of the microbial community (non-core members) over the course of fruit development and storage periods. To exclude the influence of abundance of the dominant core ASVs, we used Sorenson pairwise dissimilarity (β_SOR_), a β-diversity metric that is independent of species abundance, as well as richness variance (unlike the Bray–Curtis dissimilarity previously used to describe community succession), to explain the variation in microbial composition over time. We first calculated β_SOR_, and then partitioned it to discriminate turnover (Simpson dissimilarity; β_SIM_) from nestedness (β_SNE_) – the two antithetic processes that reflect species replacement and species loss, respectively. A mantel test revealed that the temporal distance (fruit developmental stages and storage periods) exhibited a significant association with both the turnover and nestedness components of bacterial (β_SOR_: *R^2^* = 0.59, *p* = 0.001; β_SIM_: *R^2^* = 0.36, *p* = 0.001; β_SNE_: *R^2^* = 0.40, *p* = 0.001) and fungal community variations (β_SOR_: *R^2^* = 0.57, *p* = 0.001; β_SIM_: *R^2^* = 0.35, *p* = 0.001; β_SNE_: *R^2^* = 0.31, *p* = 0.001; Figure 5A). The overall total Sorenson’s β diversity value (β_total_ value = bacteria: 0.975; fungi: 0.962) was almost entirely contributed by the turnover component measured as the Simpson dissimilarity value (β_turnover_ value-bacteria: 0.959; fungi: 0.949), as compared to the nestedness component measured as nestedness, the resultant fraction of the Sorenson dissimilarity value (β_nestedness_ value-bacteria: 0.015; fungi: 0.013; Figure 5B). These data indicate that changes in the identities of the microbial taxa that are present through succession is driven mostly by turnover. In contrast, nestedness plays only a minor role in carposphere microbiome assembly and dynamics. Since turnover was the dominant component underlying β-diversity during succession in both bacteria and fungi, we performed a distance-based test for homogeneity in multivariate dispersions (PERMDISP) to gauge the strength and timing of turnover using the β_SIM_ across the developmental stages and storage periods and visualized the results in PCoA plots. Results revealed strong turnover in species identities among the samples from the three developmental stages (fruitlet, maturation, and harvest) and little or no turnover in samples after harvest during the three time points sampled during storage (Figure 5C).

### Pattern of universality in the carposphere microbiome

**Figure 6:**
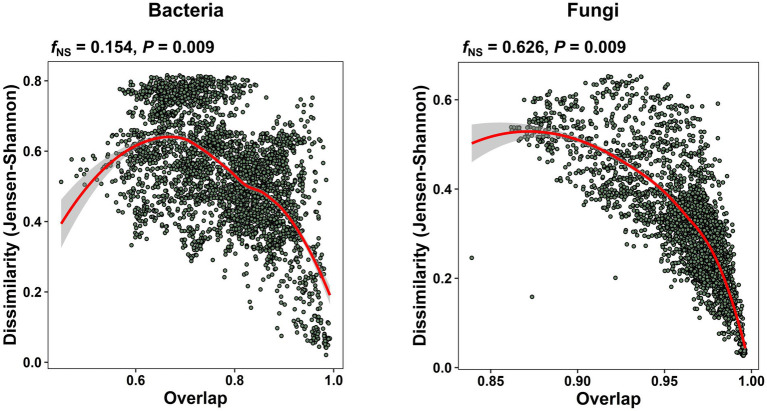
Universal ecological dynamics of the apple carposphere microbiome. Dissimilarity-overlap curves (DOC) for all bacterial and fungal samples of the apple carposphere microbiome showing significant negative slopes (*p* = 0.009). For DOCs, the overlap and dissimilarity based on root Jensen–Shannon divergence of sample pairs were calculated and each sample pair was represented as a point in the dissimilarity–overlap plane. A nonparametric regression and bootstrap sampling was performed to calculate the DOC and its confidence interval. The DOCs are indicated in red. The fraction of negative slope (*f*_NS_) is the fraction of data points in the interval where the DOC shows a negative slope and supports the level of universality. Higher *f*_NS_ value was observed in the fungal community than the bacterial community suggesting stronger level of universality in the former than the later although both were significant.

In our study of the apple carposhpere microbiome we further addressed the question of whether the carposphere microbiome assemblage follows an underlying universal ecological dynamics model. In microbial systems where universality exist, the compositional variation between sample groups mainly originates from differences in the sets of colonizing species, as opposed to individual dynamics where the samples exhibit a high degree of variability in both community assemblages and abundance profiles. We re-normalized dissimilarity (root Jensen–Shannon divergence) between all our samples for just the shared ASVs and plotted it against the overlap of the community assemblages, obtained from the relative abundances of the shared ASVs. A nonparametric regression and bootstrap sampling was performed to calculate the dissimilarity–overlap curve (DOC) and its confidence interval. A flat DOC is expected in case of individual dynamics. When a DOC displays a characteristic negative slope in the high-overlap region, universality is supported with inter-taxa interactions and the level of support is determined by the fraction of the negative slope (*f*_NS_), which is the fraction of pairwise comparisons found where the DOC slope is negative. The DOCs from the present data exhibited significant negative slopes (bacteria: *p* = 0.009; fungi: *p* = 0.009) with *f*_NS_ values of 15.4% for the bacterial community comparisons and 62.6% for the fungal community comparisons (Figure 6). To validate the universality on a larger scale, we applied the DOC analysis to a data set of from our previous study on apple fruit microbiomes from multiple geographical locations and fruit tissue types (Abdelfattah et al., 2021). We observed significant negative slopes in overall fungal community comparisons (*f*_NS_ = 48.7%, *p* = 0.009) but not significant in bacterial community comparisons (*f*_NS_ = 11.5%, *p* = 1.0; Supplementary Figure 9A). Interestingly, the level of support for universality in the fruit fungal community comparisons was highest in peel tissues (*f*_NS_ = 36.7%), followed by tissues from the calyx end (*f*_NS_ = 23.1%), and tissues from the stem end (*f*_NS_ = 18.5%; Supplementary Figure 9B).

### Cross-domain and microbe-microbe interactions in the apple carposphere

**Figure 7:**
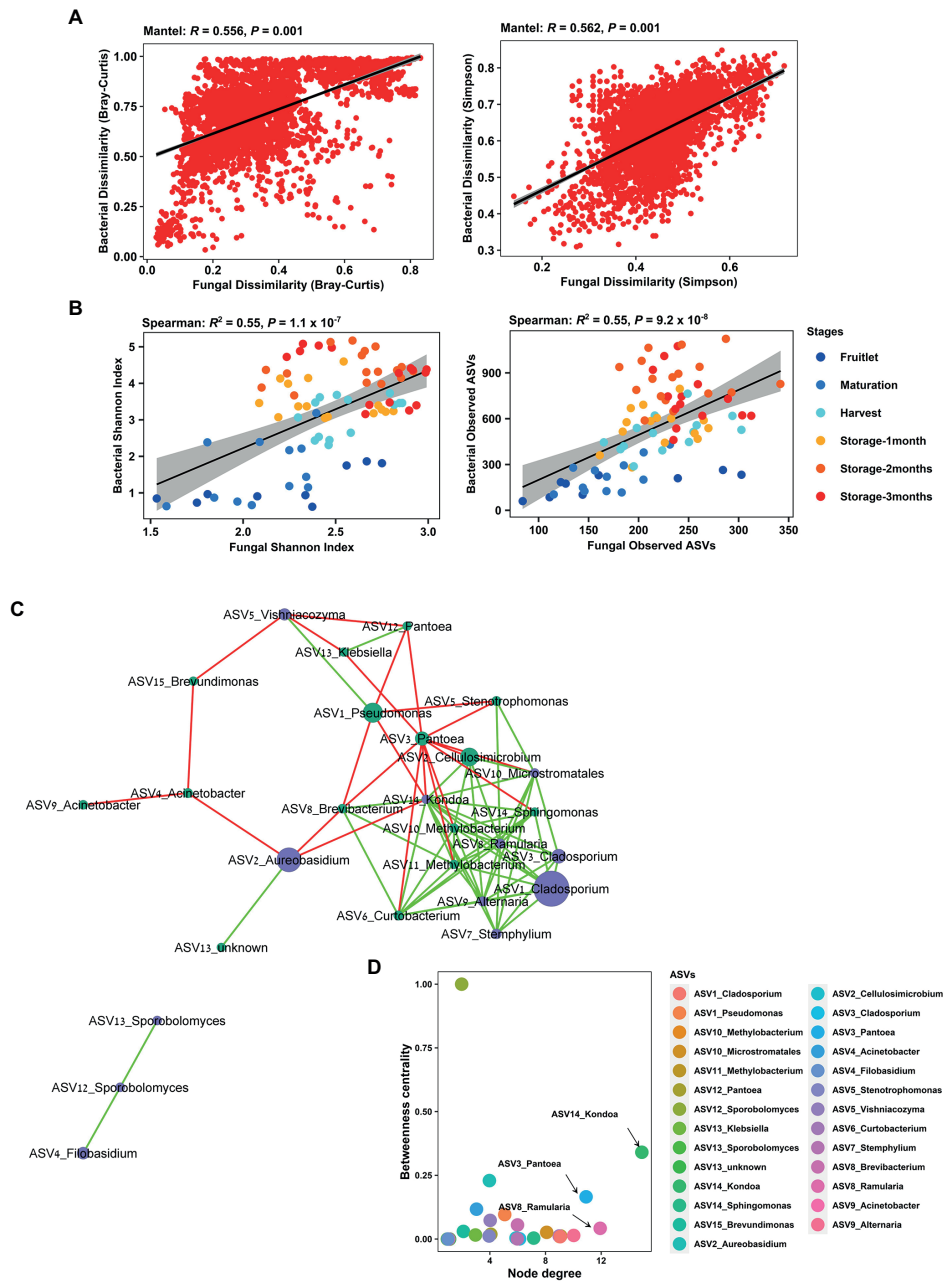
Cross-kingdom correlations between bacterial and fungal communities. **(A)** Mantel tests showing strong correlation of bacterial and fungal Bray–Curtis dissimilarity and Simpson pairwise dissimilarity comparisons across all the samples in the apple carposphere microbiome. **(B)** Significant correlation of bacterial and fungal community richness (Observed ASVs) and Shannon diversity indices across all the samples in the apple carposphere microbiome. **(C)** Co-occurrence network generated in CoNet and visualized in Cytoscape displayed significant strong positive and negative co-occurring relationships between dominant fungal and bacterial ASVs. Node colors represent ASVs from bacterial (green) and fungal kingdoms (blue). Green edges represent copresence (positive correlation) and red edges represent coexclusion (negative correlation) between relative abundance profiles indicated by the size of the nodes. Significance was estimated from multiple metrics including Spearman correlation, Pearson correlation, Bray–Curtis dissimilarity and Kullback–Leiber divergence. **(D)** Keystone taxa analysis-Betweenness centrality vs. node degree of all ASVs in the cross-domain network of the apple carposphere microbiome. Nodes with high degree represent putative hub taxa in the network and nodes with high betweenness centrality represent potential key connector species. Both these measures are indicators for potential keystone species. The taxa *Kondoa*, *Ramularia*, and *Pantoea* may act as potential keystone species in the apple carposphere microbiome.

Strong cross-domain interactions were observed, based on Mantel correlations between the bacterial and fungal community, using distance matrices based on both Bray–Curtis dissimilarity (*R* = 0.56, *p* = 0.001), where taxa abundance and richness are considered, and Simpson dissimilarity (*R* = 0.56, *p* = 0.001), where the variance due to abundance and richness was removed (Figure 7A). This cross-domain association was also supported by the detection of significant positive correlations (Spearman’s) in richness (Observed ASVs, *R* = 0.55, *p* = 9.2 × 10^−8^) and within sample diversity (Shannon Index, *R* = 0.55, *p* = 1.1 × 10^−7^) between the bacterial and fungal communities (Figure 7B). Furthermore, we generated a correlation network (27 nodes, 79 edges, network density: 0.279; characteristic path length: 2.181) to check for potential associations at the genera level, and the resulting network revealed significant co-occurrences among dominant bacterial and fungal ASVs (Figure 7C). The taxa involved comprised 15 bacterial and 12 fungal ASVs connected by 56 co-presence and 23 co-exclusion associations, and included plant pathogen genera, such as *Ramularia*, *Alternaria*, and *Stemphylium*, and biocontrol agents, such as *Aureobasidium*, *Vishniacozyma,* and *Filobasidium*. The genera *Kondoa*, *Ramularia,* and *Pantoea* were identified as putative hub taxa, based on both a high node degree and betweennesss centrality (Figure 7D).

## Discussion

Epiphytic and endophytic microbiota are integral components of the carposphere in horticultural crops and may play a contributing role in determining produce quality and shelf life. The drivers involved in the assemblage of fruit-associated microbial community, however, are just beginning to be investigated. Our principal objective in the present study was to elucidate the patterns of apple epiphytic carposphere microbiome assembly and dynamics during fruit development in the orchard and in storage utilizing three commercial apple cultivars. Our findings revealed that the structure and assembly of the apple carposphere microbiome is strongly influenced by the stage of fruit development and that the fruit genotype also exerts a significant influence (Figure 2). A significant effect of host genotype was previously reported for apple fruit endophytic microbiota (Liu et al., 2018). This may be attributed to the fact that endophytic microbes are sheltered from external factors and environmental fluctuations, and because the plant host is able to exert more control over their colonization and dynamics, in a genotype-specific manner. Our finding that the host genotype can also influence, albeit to a lesser extent, the fruit epiphytic community in this study is remarkable, considering that epiphytes are exposed to numerous external factors. The genotype effect observed on the fruit epiphytic community could possibly be attributed to differences in the degrees of host facilitative priority effects. Indeed, evidences of host genotype modulated facilitative priority effects on microbial assemblies has been reported in plant–microbe interactions (Halliday et al., 2020; Leopold and Busby, 2020).

Despite seeing strong microbial community succession across fruit stages, we observed that a fraction of the total observed ASVs comprised a core successional microbiome (15 out of 17,655 bacterial ASVs and 35 out of 3,224 fungal ASVs) and dominated in their abundance relative to other taxa (non-core members) and persisted throughout the stages of fruit development and storage (Figures 3, 4). Indeed, most of the core bacterial and genera identified by this approach like *Pseudomonas*, *Pantoea*, *Methylobacterium*, *Cladosporium*, *Aureobasidium*, *Filobasidium*, *Vishniacozyma*, and *Alternaria* among others were also frequently isolated from the fruit washings using traditional culture methods (data not shown). We suggest that this core successional microbiome could be prioritized in future studies, due to the fact that they were shared in all three cultivars and persisted throughout the different stages of fruit development and after harvest when the apples were placed in storage. Notably, these taxa included all the core groups identified in our previous global (geographically) study of the apple microbiome (Abdelfattah et al., 2021). The core taxa belonging to *Pseudomonas*, *Pantoea*, *Vishniacozyma*, *Filobasidium,* and *Aureobasidium* were differentially abundant during early fruit stages (fruitlet and maturation). These taxa contain species with demonstrated biocontrol activity against fungal pathogens that infect apples (Filonow et al., 1996; Ippolito et al., 2000; Nunes et al., 2001; Lutz et al., 2013; Wallace et al., 2017), while other core taxa, such as *Cladosporium*, *Ramularia*, *Stemphylium,* and *Alternaria* were differentially abundant during sampled storage time points and include potential postharvest pathogens of apple fruit (Videira et al., 2015; Lutz et al., 2017). Core microbiomes across different plant species, such as *Arabidopsis thaliana,* rice, sugarcane, barley, soybean, and fruit crops, such as grapes, apple, and citrus, share common members, including *Pseudomonas*, *Methylobacterium*, *Sphingomonas,* and *Cladsporium*. The fact that a set of core taxa persist in the apple carposphere starting from early fruit development through harvest and during cold storage strongly suggest that these taxa of are well-adapted to the fruit peel of apples, regardless of environmental conditions, cultural management practices, and genotype.

Microbial assembly in plant-associated microbiomes is an ecological process that involves complex interactions among diverse groups of microbes, their host plants, and the environment. Successional patterns have been reported in a few host plants, including sorghum (Gao et al., 2019, 2020), grasses and forbs (Hannula et al., 2019), and in *Baccharis linearis* (Gazitúa et al., 2021). Those studies revealed varying contributions of turnover and nestedness in shaping the dynamics of the successional assemblages. Our study on the apple carposphere indicated that the successional events involve both turnover and nestedness processes across the different stages of fruit development and storage (Figure 5A). The contribution of turnover in the community dynamics, however, far outweighed the contribution of nestedness in both bacterial and fungal communities (Figure 5B). Our results also revealed that microbial community turnover was maximum during the fruit developmental stages with little to no turnover occurring during storage (Figure 5C). The strong turnover driving community succession in the apple carposphere may perhaps be attributed to the significant niche differences present in the orchard during fruit development and the stable and regulated conditions that occur in storage. As previously stated, available niches on the apple fruit surface are subject to constant fluctuations as fruit develops and matures, with niches expanding as the surface area in growing fruit increases, which presumably continues until harvest. During these stages, the expanding carposphere is anticipated to facilitate continuous microbial immigration (hence more richness and turnover) and allow for coexistence of taxa that can adapt to the same niche environment (HilleRisLambers et al., 2012). After harvest and during storage, the overall dispersion of the communities responsible for variances in the composition over time do not exhibit any clear and predictable patterns. Developing, ripening, and senescing apple fruit undergo a series of biochemical events, including soluble sugar accumulation, change in pH, a decline in host defense response, ethylene production, increased respiration, flesh softening, etc. (Toivonen and Brummell, 2008), and all these events potentially influence the dynamics of the fruit microbiome. Sustained low temperatures during storage can also exert a profound effect on fruit microbiome dynamics, with previous studies reporting a general decrease in diversity and fluctuations in the relative abundance of microbial taxa in stored apples (Bösch et al., 2021). As such, incorporating information of these fruit physiological parameters and their influence on the fruit microbiome will provide a more comprehensive picture of the factors shaping the ecology and dynamics of fruit-associated microbiome, and we acknowledge the lack of this information as a limitation in the present study.

The existence of an underlying universal ecological dynamic in fruit microbiomes was found (Figure 6), similar to those reported for microbiomes in humans and some agricultural-associated microbial ecosystems (Bashan et al., 2016; Van Geel et al., 2017; Verbruggen et al., 2018). Our result is supported by further analysis of fruit microbiome datasets from our previous study characterizing the apple fruit microbiomes from multiple geographical locations (Supplementary Figure 9). Notably, the level of universality was higher in fungal communities than in bacterial communities in all the datasets examined. A more stable pattern of community dynamics in fungi relative to bacteria is also supported by our result showing more stable abundance distribution patterns in core successional microbes (Figure 4A). Importantly, in regards to the practical use of biocontrol technologies is that the existence of universal ecological dynamics suggests the carposphere may respond consistently across systems (cultivars, geographic locations) to microbiome manipulations (e.g., management practices, biocontrol applications, etc.).

We observed a strong correlation between bacterial and fungal microbiomes (a cross-domain association) as indicated by their significant correlation in their community composition and diversity (Figures 7A,B). In some cases, bacteria and fungi may co-occur in syntrophic guilds where, besides other interactions, the members interact mutually by producing metabolites for others to use (Hoffmann et al., 2013). We also generated a co-occurrence network from across all samples to investigate microbe-microbe interaction dynamics and revealed several individual bacterial and fungal taxa, including putative plant pathogens as well as biocontrol agents, with significant positive or negative co-occurrences among them (Figure 7C). This network approach may be insufficient for interpreting species interactions and does not indicate causal relationships (Röttjers and Faust, 2018). Identifying these correlations and hub microorganisms through co-occurrence networks, however, provides a useful starting point for experimental studies aimed at testing relationships and constructing synthetic communities to manipulate fruit–microbiomes.

In summary, we demonstrated that fruit developmental stage and the length of storage significantly shape assemblages of microbial communities on fruit surface, while fruit genotype also plays a role in the overall assembly and dynamics. We observed strong succession in microbial communities of the apple carposphere that is strongly driven by turnover events during the different fruit developmental stages. The underlying ecological dynamics of the apple carposphere largely follow a universal model. We found that a set of core taxa persist throughout the stages of fruit development and after harvest and identified specific taxa, including known biocontrol agents of plant pathogens, that were differentially abundant during the different stages of fruit development. Further studies should investigate core microbial members for direct functional interactions with fruit pathogens and their interactions with the resident microbiome.

## Materials and methods

### Experimental set-up and sampling

In order to minimize variations due to effects of environmental conditions, we selected three apple cultivars—“Royal Gala,” “Granny Smith” and “Golden Delicious,” that were planted at the Matityahu Experimental Farm, Agricultural Research Organization (ARO), Northern Israel (3380400400 N, 3582700400 E, altitude 667 m). The three cultivars were planted in the same plot of the orchard where several other apple cultivars in separate rows were also planted with 3-m row gaps and each row consisted of 35–40 individual trees. Three rows, one each of the three cultivars were tagged for use in this study. The cultivars “Granny Smith” and “Golden Delicious” have different genetic lineages arising from chance seedlings while “Royal Gala” is a product of traditional breeding between “Kidds Orange Red” and “Golden Delicious.” All trees of the three cultivars were the same age (5 years) and under the same maintenance program. The apple trees received fungicidal and insecticidal sprays against apple scab, powdery mildew, codling moth, fruit fly and mite during the previous seasons (Supplementary Table 1) but their application was avoided during the duration of this experiment (2019 season).

Sampling of fruit was done from all the selected trees during three fruit developmental stages *viz.* at fruitlet stage (50–60 days after anthesis; DAA), at maturation stage (110–120 DAA) and at harvest stage (150–170 DAA), followed by another three times at monthly intervals from the harvested fruit lot during cold-storage periods (1°C; Figure 1). Details of dates of sampling, number of replicates for each sampling time and average fruit sizes during different sampling times are summarized in Supplementary Table 2. These stages were selected to encompass the major physiological stages of fruit during development in the field and storage after harvest. The onset of anthesis of the three cultivars occurred around the same time (between March 20 and 30, 2019). The fruits from all the three cultivars were collected at the same date for fruitlet stage on 29th May 2019 (50–60 days after anthesis; DAA) and for maturation stage on 28th July 2019 (110–120 DAA). Due to differences in ripening times of the three cultivars, harvesting was done on different dates (“Royal Gala” on 27th August 2019, “Golden Delicious” on 3rd September 2019 and “Granny Smith” on 16th September 2019). Briefly, 3–5 fruits from each tree were picked manually using sterile gloves during fruitlet and maturation stages and 20–30 fruits from each tree during harvest stage. At each picking time, fruits from all the trees for each cultivar were pooled, collected in sterile corrugated fiberboard boxes, marked and transported immediately to the laboratory for sampling. All the fruits picked at fruitlet and maturation stages were immediately used for sampling while enough fruits were picked at harvest stage to be sampled immediately and also after different storage times. For this, harvested fruits of the three cultivars were all stored together in the same cold-storage room (1°C, 85% relative humidity), in separate containers. Prior to sampling, these stored fruits were removed from storage and placed at room temperature for 5 h and allowed to warm. Weather information (temperature and humidity) during the course of the experiment (from March 2019 to October 2019) is shown in Supplementary Figure 1.

### DNA extraction and sequencing of 16S rRNA and ITS genes

Epiphytic microorganisms were obtained by swabbing the entire fruit surface using sterile cotton tips moistened with phosphate buffered saline (PBS). Swabbing was done on 20 fruits (using a swab for each fruit and later pooled) to represent one sample replicate for all sampling stages and cultivars. A fixed number of 20 fruits each per replicate was used to obtain the samples from each cultivar and sampling points in order to account for variation arising from the expanding fruit surface area as the fruit developed. This way, using different set of 20 fruits for each sampling replicate, a total of 1,640 fruits were used to obtain a total of 82 samples with ≥3 replicates per sample depending on the availability of fruits. The cotton swabs were collected in 50 ml falcon tubes containing 20 ml of PBS, gently shaken (150 rpm, 10-min) in a rotary shaker, sonicated for 5-min in a water bath sonication followed by a 30-s vortex. After aseptically removing the cotton tips, the microbial suspension obtained was used to extract DNA by using the Promega DNA Purification kit according to the manufacturer’s instructions. To check for contamination introduced during extraction, elution buffer and specimen-free DNA isolations were performed and used as negative controls.

Extracted DNA was used for amplicon PCR reactions to amplify the bacterial 16S rRNA region using the 515F/806R primer set, and the fungal internal transcribed spacer (ITS) region using the ITS3/KYO2 primer set along with peptide nucleic acid (PNA) clamps to block the PCR amplification of apple plastid and mitochondrial sequences (Caporaso et al., 2011; Toju et al., 2012). PCR conditions were performed as described in our previous studies (Abdelfattah et al., 2021). Library preparation following amplification was done as specified in the Illumina 16S Metagenomic Sequencing Library Preparation guide precisely as outlined. Sterile water and empty wells were sequenced as negative controls. Subsequent library size, quality, and confirmation of the absence of adapter dimers were done on an Agilent 2100 Bioanalyzer (Agilent). Paired-end sequencing of amplicons was conducted on an Illumina MiSeq (Illumina) sequencer with a V3 600-cycle Reagent Kit (Illumina).

### Data processing and analysis

Demultiplexed forward and reverse reads of both 16S rRNA and 1TS gene sequences were trimmed, merged, denoised, and chimeras removed for quality control using default parameters in DADA2 (Callahan et al., 2016) as integrated in QIIME2 (Bolyen et al., 2019). The taxonomies of the high-resolution amplicon sequence variants (ASVs) obtained were performed using the GreenGenes and UNITE databases for 16S rRNA reads (DeSantis et al., 2006) and ITS reads (Abarenkov et al., 2010) respectively. Negative controls were used to identify the contaminant sequences and removed before any analyses were performed. A total 17,655 bacterial ASVs (11,540,568 reads) and 3,224 fungal ASVs (13,110,514 reads) were obtained. To account for differences in sequencing depth, the ASV feature tables were rarified to the depth of the smallest sample (31,899 for 16S rRNA and 84,687 for ITS) and all downstream analyses were performed on this rarefied ASV tables.

Rarefaction curves for both 16S rRNA and ITS reads were constructed and confirmed the minimum depths used were sufficient to reach saturations of diversity in both bacterial and fungal communities (Supplementary Figure 2). Bray–Curtis dissimilarities were calculated and subjected to principal coordinate analysis (PCoA) using the ordination function in the phyloseq package in R v3.5.1 to visualize the variations in microbial community compositions between groups. In addition, permutational analysis of variance (PERMANOVA) was carried out to assess the effect of fruit stage and genotype on the microbial community variation detected by Bray–Curtis dissimilarities using the adonis function in the vegan package. To test for homogeneity among microbial communities, β-dispersions using Bray–Curtis and Simpson dissimilarities were explored by the betadisper function in the vegan package in R.

The α-diversity indices (Observed ASVs and Shannon Index) were calculated using the estimate richness function in the phyloseq package. Statistically significant differences in diversity metrics were identified using the nonparametric Kruskal–Wallis for comparisons between all the groups, and pair wise comparisons between samples were made using the Wilcoxon’s test.

To visualize the relative abundances of ASVs, bar plots and line graphs were constructed using the ggplot2 package. The core microbiome was calculated based on ASVs present in 95% of the investigated samples using core function in the microbiome package. Linear discriminant analysis (LDA) effect size (LEfSe; Segata et al., 2011) that utilizes a combination of Kruskal–Wallis test and pairwise Wilcoxon rank-sum test with linear discriminant analysis (LDA > 4), was used to determine a ASVs that best characterize each fruit developmental stage and storage period.

Bray–Curtis dissimilarities between sample pairs and Euclidean dissimilarities were used to construct distance matrices of temporal distances (fruit stages) and Mantel tests were performed to explore the correlations between these matrices. Sorensen pair wise dissimilarity (β_SOR_) distances between sample pairs were measured based on presence/absence data (to remove the richness variation), and then partitioned into those contributed from turnover (Simpson pair wise dissimilarity; β_SIM_) and nestedness-resultant dissimilarity (β_NES_) using the betapart package, followed by Mantel tests to explore the correlations between these matrices with the temporal distance (fruit stages).

To assess the universality in ecological dynamics across fruit microbial communities, we used the dissimilarity overlap curve (DOC) approach using the DOC package. Briefly, the DOC was constructed by plotting, for each possible sample pair of microbial communities, the dissimilarity on the *y*-axis (root Jensen–Shannon divergence, calculated from only the ASVs shared by the two communities) against the fraction of ASVs that overlap on the *x*-axis. Universality is supported where the DOC dips as the overlap grows, and this level of support is proportional to the fraction of pairwise comparisons where the DOC slope is negative (termed the fraction of negative slope, *f*_NS_). For the smoothed DOC curve, the initiation of negative slope signifies the median of initiation of negative slopes obtained from DOCs of 1,000 bootstrapped data sets.

To explore microbe-microbe correlations, we selected ASVs with relative abundances >1% identified across all the samples, merged the bacterial and fungal ASVs and constructed a co-occurrence network using the CoNet (Faust and Raes, 2016) application (v.1.1.1.beta) implemented in Cytoscape (v.3.7.2; Shannon et al., 2003). Briefly, Pearson’s correlations, Spearman’s correlations, Bray–Curtis dissimilarity and Kullback–Leibler divergence, were performed and used to create an initial network. The edgeScores randomization function was then employed to perform 100 row-wise permutations with 1,000 highest and lowest scoring edges retained. The reboot renormalization function was then used to check for compositional bias to construct a merged final network based on a score distribution of 100 bootstrap iterations. Significance of the correlations was calculated with Brown’s method and Benjamini–Hochberg FDR method was used for correcting multiple comparisons. The network was finally visualized using an organic layout in Cytoscape (v3.7.2).

## Data availability statement

The sequencing data generated from 16S rRNA and ITS genes in this study has been deposited to the NCBI SRA database under the accession number Bioproject ID: PRJNA770498.

## Author contributions

VZ, AK, AB, and SD designed the study. VZ, AK, AB, AA, VS, SS, OF, RB, SF, SW, MW, and SD contributed in conducting the experiment. VZ, AK, and AB performed the analyses. VZ and SD wrote the first draft. All authors contributed to the article and approved the submitted version.

## Funding

This research was supported by U.S. – Israel Binational Agricultural Research and Development Fund (BARD) IS-5455-21. Additional support was provided by Agriculture and Food Research Initiative Competitive Grant no. 2018-07366 from the USDA National Institute of Food and Agriculture to SW.

## Conflict of interest

The authors declare that the research was conducted in the absence of any commercial or financial relationships that could be construed as a potential conflict of interest.

## Publisher’s note

All claims expressed in this article are solely those of the authors and do not necessarily represent those of their affiliated organizations, or those of the publisher, the editors and the reviewers. Any product that may be evaluated in this article, or claim that may be made by its manufacturer, is not guaranteed or endorsed by the publisher.

## Supplementary material

The Supplementary material for this article can be found online at: https://www.frontiersin.org/articles/10.3389/fmicb.2022.928888/full#supplementary-material
