# Supplementary material for: Assembly and dynamics of the apple carposphere microbiome during fruit development and storage
Source: Front Microbiol. 2022 Aug 9;13:928888. doi: 10.3389/fmicb.2022.928888 (PMC9395710; doi:10.3389/fmicb.2022.928888)
Supplement: Supplementary file 1 [file Data_Sheet_1.docx]

## *Supplementary Material*

The following supporting information is available for this article:

**Supplementary Table 1: Insecticides and fungicides used in the apple orchard during earlier seasons in the experimental field.**

| **Target pests/ diseases** | **Insecticides/ Fungicides** | **Dosage** |
| --- | --- | --- |
| Mite | Meteor (Fenpyroximate) | 0.10 % |
| Weevil, Mite | Vectra (Abamectin) | 0.04 % |
| Mite | Lintex (Cyhexatin) | 0.06 % |
| Apple moth | Armada (Spinetoram + Methoxyfenozide) | 0.03 % |
| Apple Moth | Tsabar (Lufenuron) | 0.10 % |
| Aphid, Weevil | Mavrik (Tau-fluvalinate) | 0.05 % |
| Powdery mildew, Apple scab | Phantom (Trifloxystrobin) | 0.02 % |
| Powdery mildew, Apple scab | Merpan (Captan) | 0.15 % |
| Powdery mildew, Apple scab | Skipper (Difenoconazole) | 0.02 % |

**Supplementary Table 2: Sample collection dates and average weights of fruits at different stages.**

| **Fruit stages** | **Date of collection of samples** | | | **Average weight of fruit (g)** | | |
| --- | --- | --- | --- | --- | --- | --- |
|  | **Royal Gala** | **Golden Delicious** | **Granny Smith** | **Royal Gala** | **Golden Delicious** | **Granny Smith** |
| **Fruitlet** | 29^th^ May 2019  (n= 3) | 29^th^ May 2019  (n= 3) | 29^th^ May 2019  (n= 3) | 13.28±1.25 | 8.93±1.12 | 14.30±2.11 |
| **Maturation** | 28^th^ July 2019  (n= 4) | 28^th^ July 2019  (n= 4) | 28^th^ July 2019  (n= 4) | 56.77±2.44 | 45.31±3.88 | 61.27±3.87 |
| **Harvest** | 27^th^ Aug. 2019  (n= 5) | 3^rd^ Sept. 2019  (n= 5) | 16^th^ Sept. 2019  (n= 4) | 155.41±3.67 | 145.30±6.56 | 175.32±4.65 |
| **Storage-1 month** | 26^th^ Sept. 2019  (n= 5) | 3^rd^ Oct. 2019  (n= 5) | 17^th^ Oct. 2019  (n= 5) | 154.23±4.46 | 140.55±4.28 | 175.25±5.20 |
| **Storage-2 months** | 27^th^ Oct. 2019  (n= 4) | 3^rd^ Nov. 2019  (n= 5) | 17^th^ Nov. 2019  (n= 8) | 153.14±4.23 | 141.41±5.14 | 174.23±3.87 |
| **Storage-3 months** | 26^h^ Nov. 2019  (n= 5) | 2^nd^ Dec. 2019  (n= 5) | 16^th^ Dec. 2019  (n= 5) | 156.81±5.98 | 139.26±3.45 | 173.67±4.44 |

*Values in parenthesis indicate the number of samples during each collection. A sample consisted of swabs from 20 fruit each.





**Supplementary Figure 1:  Seasonal dynamics of temperature and rainfall at the site and time of the experiment.**

**
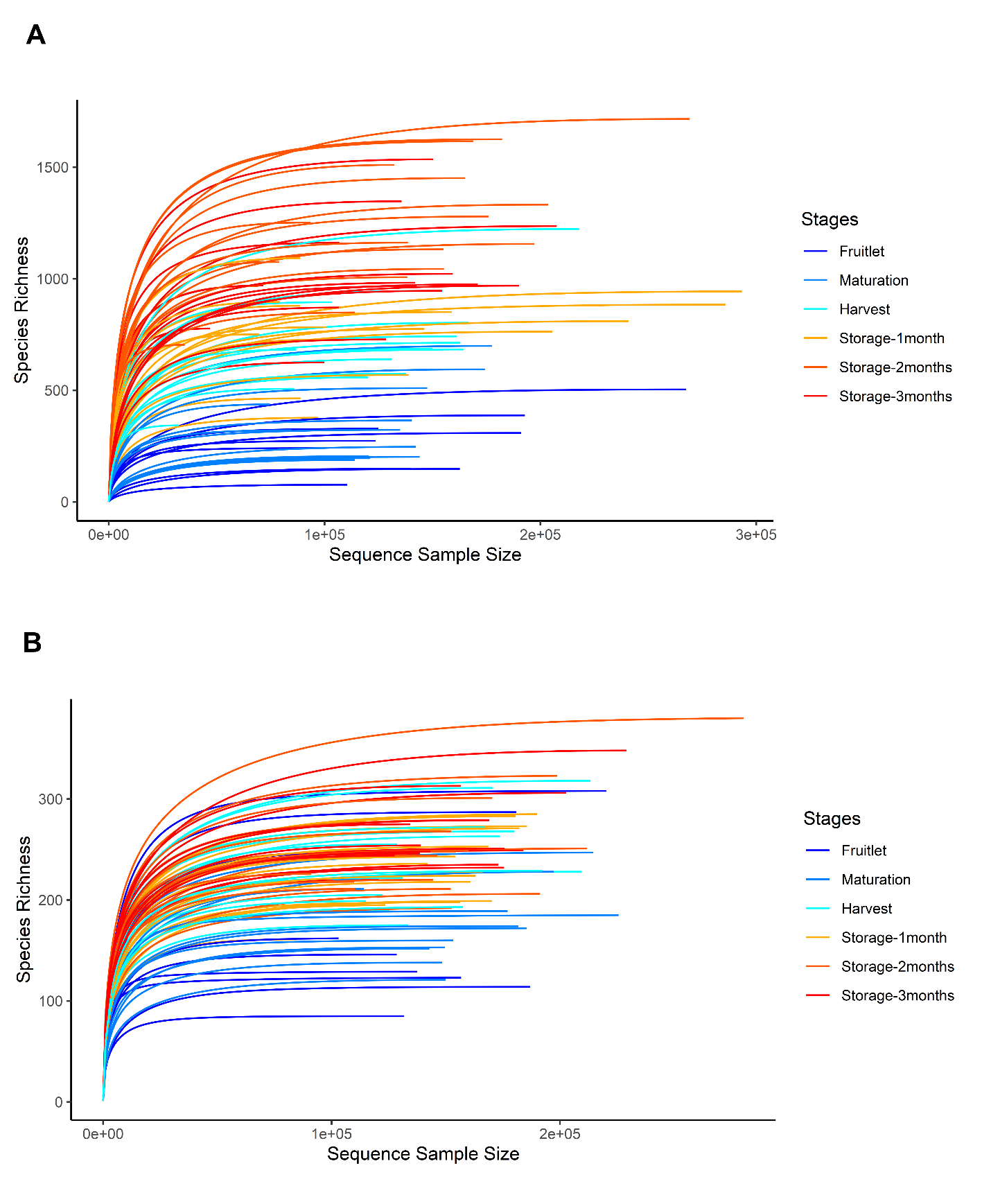
**

**Supplementary Figure 2: Rarefaction plots of detected 16S (a) and ITS (b) amplified sequence variants (ASVs) by sampling depth.**





**Supplementary Figure 3:** **Fruit developmental stages and storage significantly shapes the apple carposphere microbiome.**

Principal coordinate (PCoA) analysis of bacterial and fungal community based on Bray–Curtis dissimilarity with permutational analysis of variance (PERMANOVA) showed significant association of both bacterial and fungal community composition with fruit developmental stages and storage (*P* = 0.001).


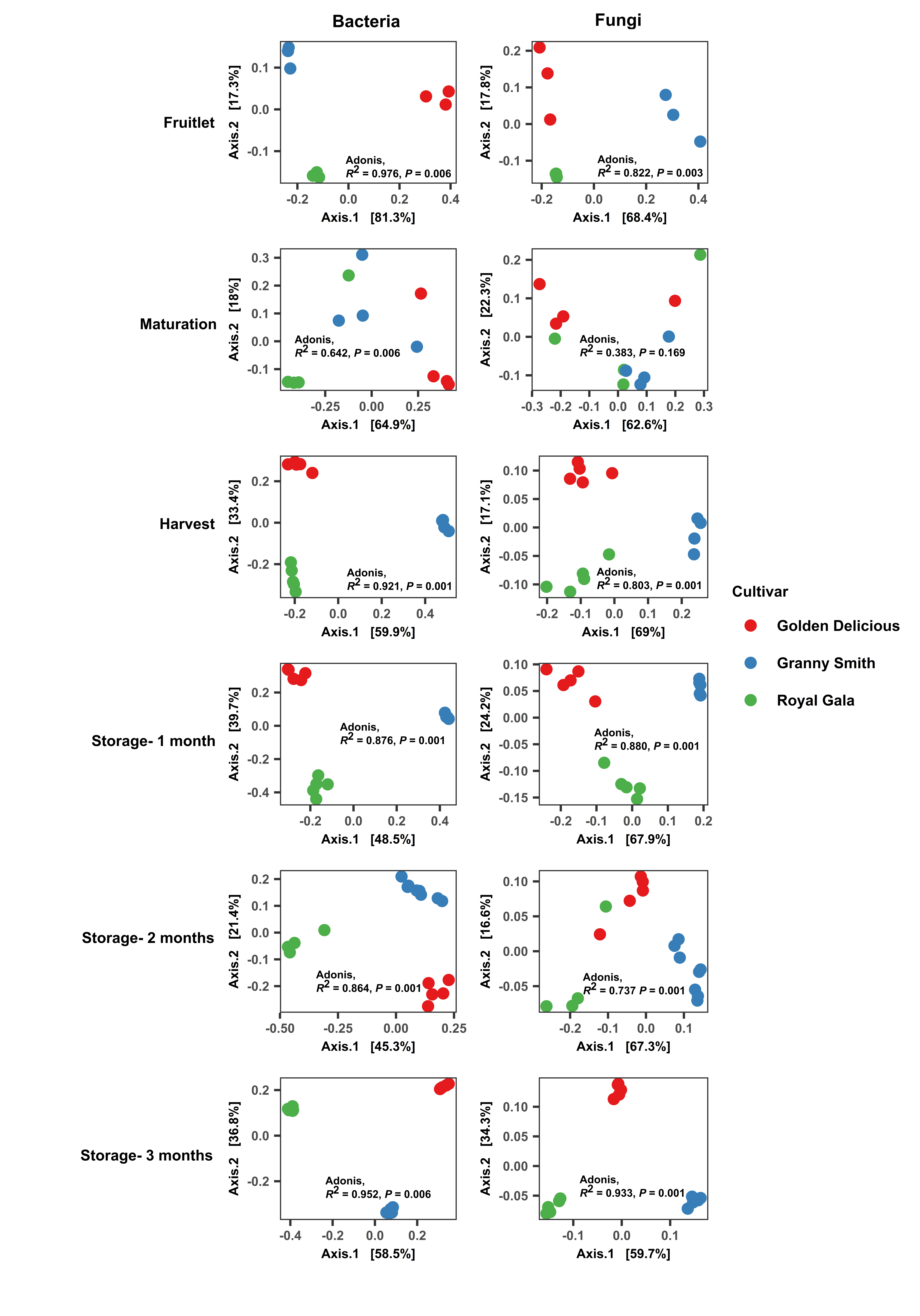


**Supplementary Figure 4:** **Microbiome composition differs between cultivars at different fruit developmental stages and storage periods in the apple carposphere.**

Principal coordinate (PCoA) analysis of bacterial and fungal community based on Bray–Curtis dissimilarity with permutational analysis of variance (PERMANOVA) showed significant differences between the three apple cultivars at different fruit developmental stages and storage periods (*P* = 0.001).

**Supplementary Table 3. Pairwise comparisons (Adonis) of epiphytic apple microbiome composition between cultivars at different stages of the fruit, based on Bray-Curtis dissimilarity. *P*-values were calculated using the pairwiseAdonis function in vegan and corrected for multiple testing using the Bonferroni method.**

| **Fruitlet** | **Bacteria** | | | **Fungi** | | |
| --- | --- | --- | --- | --- | --- | --- |
|  | ***R*^2a^** | ***P* value^b^** | ***P* adjusted^b^** | ***R*^2a^** | ***P* value^b^** | ***P* adjusted^b^** |
| Royal Gala vs Granny Smith | 0.766 | 0.1 | 0.3 | 0.835 | 0.1 | 0.3 |
| Royal Gala vs Golden Delicious | 0.923 | 0.1 | 0.3 | 0.404 | 0.1 | 0.3 |
| Granny Smith vs Golden Delicious | 0.909 | 0.1 | 0.3 | 0.768 | 0.1 | 0.3 |
| **Maturation** |  |  |  |  |  |  |
|  | ***R*^2a^** | ***P* value^b^** | ***P* adjusted^b^** | ***R*^2a^** | ***P* value^b^** | ***P* adjusted^b^** |
| Royal Gala vs Granny Smith | 0.354 | 0.056 | 0.168 | 0.209 | 0.208 | 0.624 |
| Royal Gala vs Golden Delicious | 0.733 | 0.027 | 0.081 | 0.132 | 0.498 | 1 |
| Granny Smith vs Golden Delicious | 0.425 | 0.071 | 0.213 | 0.406 | 0.068 | 0.204 |
| **Harvest** |  |  |  |  |  |  |
|  | ***R*^2a^** | ***P* value^b^** | ***P* adjusted^b^** | ***R*^2a^** | ***P* value^b^** | ***P* adjusted^b^** |
| Royal Gala vs Granny Smith | 0.907 | 0.01 | 0.03 | 0.696 | 0.009 | 0.027 |
| Royal Gala vs Golden Delicious | 0.538 | 0.005 | 0.015 | 0.317 | 0.026 | 0.078 |
| Granny Smith vs Golden Delicious | 0.684 | 0.01 | 0.03 | 0.662 | 0.009 | 0.027 |
| **Storage-1month** |  |  |  |  |  |  |
|  | ***R*^2a^** | ***P* value^b^** | ***P* adjusted^b^** | ***R*^2a^** | ***P* value^b^** | ***P* adjusted^b^** |
| Royal Gala vs Granny Smith | 0.817 | 0.008 | 0.024 | 0.677 | 0.009 | 0.027 |
| Royal Gala vs Golden Delicious | 0.725 | 0.011 | 0.033 | 0.529 | 0.013 | 0.039 |
| Granny Smith vs Golden Delicious | 0.831 | 0.008 | 0.024 | 0.890 | 0.006 | 0.018 |
| **Storage-2months** |  |  |  |  |  |  |
|  | ***R*^2a^** | ***P* value^b^** | ***P* adjusted^b^** | ***R*^2a^** | ***P* value^b^** | ***P* adjusted^b^** |
| Royal Gala vs Granny Smith | 0.561 | 0.003 | 0.009 | 0.503 | 0.003 | 0.009 |
| Royal Gala vs Golden Delicious | 0.476 | 0.013 | 0.039 | 0.468 | 0.009 | 0.027 |
| Granny Smith vs Golden Delicious | 0.324 | 0.001 | 0.003 | 0.306 | 0.004 | 0.012 |
| **Storage-3months** |  |  |  |  |  |  |
|  | ***R*^2a^** | ***P* value^b^** | ***P* adjusted^b^** | ***R*^2a^** | ***P* value^b^** | ***P* adjusted^b^** |
| Royal Gala vs Granny Smith | 0.765 | 0.012 | 0.036 | 0.755 | 0.01 | 0.03 |
| Royal Gala vs Golden Delicious | 0.796 | 0.01 | 0.03 | 0.595 | 0.005 | 0.015 |
| Granny Smith vs Golden Delicious | 0.846 | 0.006 | 0.018 | 0.634 | 0.006 | 0.018 |

^a^ *R*^2^ values indicate the percent variation in community dissimilarity.

^b^Significant *P* values are highlighted in red.

**Supplementary Table 4. Pairwise comparisons (Adonis) of epiphytic apple microbiome composition between different stages of the fruit, based on Bray-Curtis dissimilarity. *p*-values were calculated using the pairwiseAdonis function in vegan and corrected for multiple testing using the Bonferroni method.**

| **Pairwise comparisons of stages** | **Bacteria** | | | **Fungi** | | |
| --- | --- | --- | --- | --- | --- | --- |
|  | ***R*^2a^** | ***P* value^b^** | ***P* adjusted^b^** | ***R*^2a^** | ***P* value^b^** | ***P* adjusted^b^** |
| Fruitlet vs Maturation | 0.177 | 0.023 | 0.345 | 0.338 | 0.001 | 0.015 |
| Fruitlet vs Harvest | 0.424 | 0.001 | 0.015 | 0.465 | 0.001 | 0.015 |
| Fruitlet vs Storage-1month | 0.404 | 0.001 | 0.015 | 0.563 | 0.001 | 0.015 |
| Fruitlet vs Storage-2months | 0.524 | 0.001 | 0.015 | 0.592 | 0.001 | 0.015 |
| Fruitlet vs Storage-3months | 0.526 | 0.001 | 0.015 | 0.624 | 0.001 | 0.015 |
| Maturation vs Harvest | 0.327 | 0.001 | 0.015 | 0.155 | 0.004 | 0.06 |
| Maturation vs Storage-1month | 0.314 | 0.001 | 0.015 | 0.254 | 0.001 | 0.015 |
| Maturation vs Storage-2months | 0.441 | 0.001 | 0.015 | 0.329 | 0.001 | 0.015 |
| Maturation vs Storage-3months | 0.438 | 0.001 | 0.015 | 0.390 | 0.001 | 0.015 |
| Harvest vs Storage-1month | 0.089 | 0.021 | 0.315 | 0.093 | 0.051 | 0.765 |
| Harvest vs Storage-2months | 0.110 | 0.005 | 0.075 | 0.127 | 0.003 | 0.045 |
| Harvest vs Storage-3months | 0.143 | 0.001 | 0.015 | 0.172 | 0.003 | 0.045 |
| Storage-1month vs Storage-2months | 0.163 | 0.001 | 0.015 | 0.048 | 0.185 | 1 |
| Storage-1month vs Storage-3months | 0.142 | 0.001 | 0.015 | 0.056 | 0.163 | 1 |
| Storage-2months vs Storage-3months | 0.120 | 0.001 | 0.015 | 0.036 | 0.336 | 1 |

^a^*R*^2^ values indicate the percent variation in community dissimilarity.

^b^Significant *P* values are highlighted in red.

**Supplementary Table 5. Pairwise comparisons (Adonis) of epiphytic apple microbiome composition between different stages of the fruit for each apple cultivars, based on Bray-Curtis dissimilarity. *P*-values were calculated using the pairwiseAdonis function in vegan and corrected for multiple testing using the Bonferroni method.**

| **Pairwise comparisons of stages** | **Bacteria** | | | **Fungi** | | |
| --- | --- | --- | --- | --- | --- | --- |
|  | ***R*^2a^** | ***P* value^b^** | ***P* adjusted^b^** | ***R*^2a^** | ***P* value^b^** | ***P* adjusted^b^** |
| **Royal Gala** | | | | | | |
| Fruitlet vs Maturation | 0.481 | 0.026 | 0.390 | 0.560 | 0.025 | 0.375 |
| Fruitlet vs Harvest | 0.854 | 0.015 | 0.225 | 0.714 | 0.015 | 0.225 |
| Fruitlet vs Storage-1month | 0.859 | 0.027 | 0.405 | 0.835 | 0.017 | 0.255 |
| Fruitlet vs Storage-2months | 0.828 | 0.028 | 0.420 | 0.832 | 0.023 | 0.345 |
| Fruitlet vs Storage-3months | 0.848 | 0.011 | 0.165 | 0.882 | 0.016 | 0.240 |
| Maturation vs Harvest | 0.533 | 0.009 | 0.135 | 0.167 | 0.228 | 1.000 |
| Maturation vs Storage-1month | 0.743 | 0.004 | 0.060 | 0.286 | 0.033 | 0.495 |
| Maturation vs Storage-2months | 0.727 | 0.036 | 0.540 | 0.203 | 0.291 | 1.000 |
| Maturation vs Storage-3months | 0.759 | 0.008 | 0.120 | 0.301 | 0.008 | 0.120 |
| Harvest vs Storage-1month | 0.767 | 0.013 | 0.195 | 0.484 | 0.008 | 0.120 |
| Harvest vs Storage-2months | 0.612 | 0.010 | 0.150 | 0.429 | 0.020 | 0.300 |
| Harvest vs Storage-3months | 0.770 | 0.007 | 0.105 | 0.511 | 0.010 | 0.150 |
| Storage-1month vs Storage-2months | 0.654 | 0.003 | 0.045 | 0.383 | 0.022 | 0.330 |
| Storage-1month vs Storage-3months | 0.731 | 0.011 | 0.165 | 0.314 | 0.038 | 0.570 |
| Storage-2months vs Storage-3months | 0.633 | 0.010 | 0.150 | 0.248 | 0.128 | 1.000 |
| **Granny Smith** | | | | | | |
| Fruitlet vs Maturation | 0.608 | 0.042 | 0.630 | 0.539 | 0.032 | 0.480 |
| Fruitlet vs Harvest | 0.969 | 0.037 | 0.555 | 0.703 | 0.028 | 0.420 |
| Fruitlet vs Storage-1month | 0.904 | 0.020 | 0.300 | 0.759 | 0.011 | 0.165 |
| Fruitlet vs Storage-2months | 0.791 | 0.005 | 0.075 | 0.679 | 0.004 | 0.060 |
| Fruitlet vs Storage-3months | 0.945 | 0.019 | 0.285 | 0.731 | 0.012 | 0.180 |
| Maturation vs Harvest | 0.772 | 0.030 | 0.450 | 0.756 | 0.025 | 0.375 |
| Maturation vs Storage-1month | 0.697 | 0.005 | 0.075 | 0.826 | 0.006 | 0.090 |
| Maturation vs Storage-2months | 0.679 | 0.002 | 0.03 | 0.574 | 0.004 | 0.060 |
| Maturation vs Storage-3months | 0.790 | 0.019 | 0.285 | 0.793 | 0.012 | 0.180 |
| Harvest vs Storage-1month | 0.902 | 0.014 | 0.210 | 0.505 | 0.006 | 0.090 |
| Harvest vs Storage-2months | 0.576 | 0.006 | 0.090 | 0.116 | 0.258 | 1.000 |
| Harvest vs Storage-3months | 0.879 | 0.014 | 0.210 | 0.337 | 0.061 | 0.915 |
| Storage-1month vs Storage-2months | 0.726 | 0.002 | 0.03 | 0.182 | 0.107 | 1.000 |
| Storage-1month vs Storage-3months | 0.867 | 0.009 | 0.135 | 0.436 | 0.022 | 0.330 |
| Storage-2months vs Storage-3months | 0.485 | 0.002 | 0.03 | 0.198 | 0.059 | 0.885 |
| **Golden Delicious** | | | | | | |
| Fruitlet vs Maturation | 0.437 | 0.062 | 0.93 | 0.500 | 0.066 | 1 |
| Fruitlet vs Harvest | 0.772 | 0.019 | 0.285 | 0.758 | 0.048 | 0.72 |
| Fruitlet vs Storage-1month | 0.881 | 0.02 | 0.3 | 0.897 | 0.054 | 0.81 |
| Fruitlet vs Storage-2months | 0.734 | 0.016 | 0.24 | 0.894 | 0.05 | 0.75 |
| Fruitlet vs Storage-3months | 0.943 | 0.022 | 0.33 | 0.919 | 0.05 | 0.75 |
| Maturation vs Harvest | 0.733 | 0.008 | 0.12 | 0.256 | 0.081 | 1 |
| Maturation vs Storage-1month | 0.832 | 0.015 | 0.225 | 0.346 | 0.085 | 1 |
| Maturation vs Storage-2months | 0.704 | 0.01 | 0.15 | 0.612 | 0.008 | 0.12 |
| Maturation vs Storage-3months | 0.899 | 0.007 | 0.105 | 0.688 | 0.013 | 0.195 |
| Harvest vs Storage-1month | 0.359 | 0.008 | 0.12 | 0.151 | 0.284 | 1 |
| Harvest vs Storage-2months | 0.391 | 0.013 | 0.195 | 0.477 | 0.007 | 0.105 |
| Harvest vs Storage-3months | 0.529 | 0.009 | 0.135 | 0.580 | 0.009 | 0.135 |
| Storage-1month vs Storage-2months | 0.376 | 0.005 | 0.075 | 0.644 | 0.01 | 0.15 |
| Storage-1month vs Storage-3months | 0.733 | 0.012 | 0.18 | 0.726 | 0.008 | 0.12 |
| Storage-2months vs Storage-3months | 0.623 | 0.01 | 0.15 | 0.426 | 0.014 | 0.21 |

^a^*R*^2^ values indicate the percent variation in community dissimilarity; ^b^ Significant *P* values are highlighted in red.


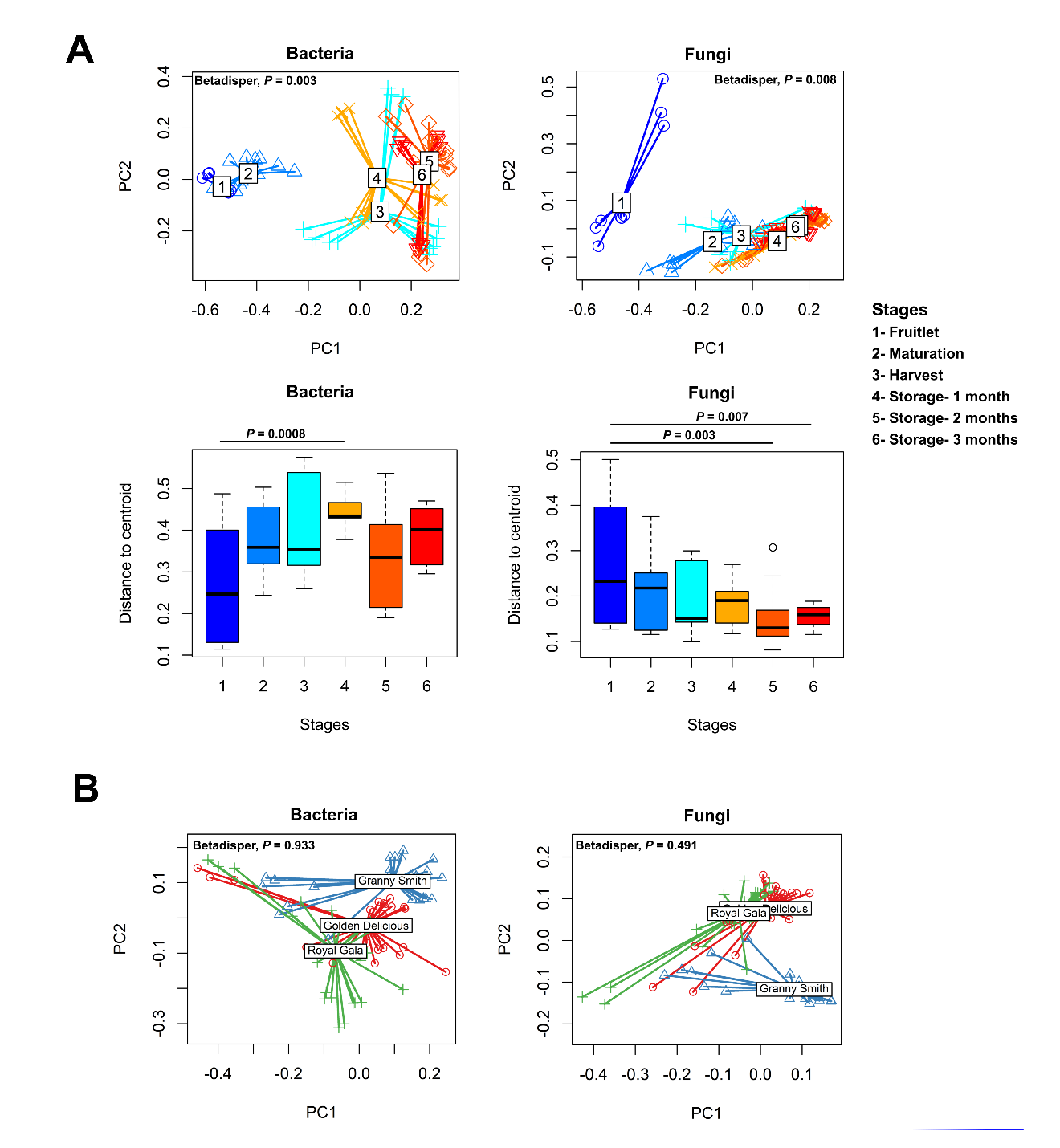


**Supplementary Figure 5: Multivariate dispersion of microbial composition during each sampling stages and in each cultivars.**

Principal coordinates analysis (PCoA) plots and bar plots of distance to centroids visualising the multivariate homogeneity of group dispersion based on Bray–Curtis distances grouped by (**a**) stages revealed significant differences in the compositional variances between the stages but (**b**) no significant differences between the samples in the three apple cultivars. *P* values in the barplots show significant differences between stages (Tukey HSD test).


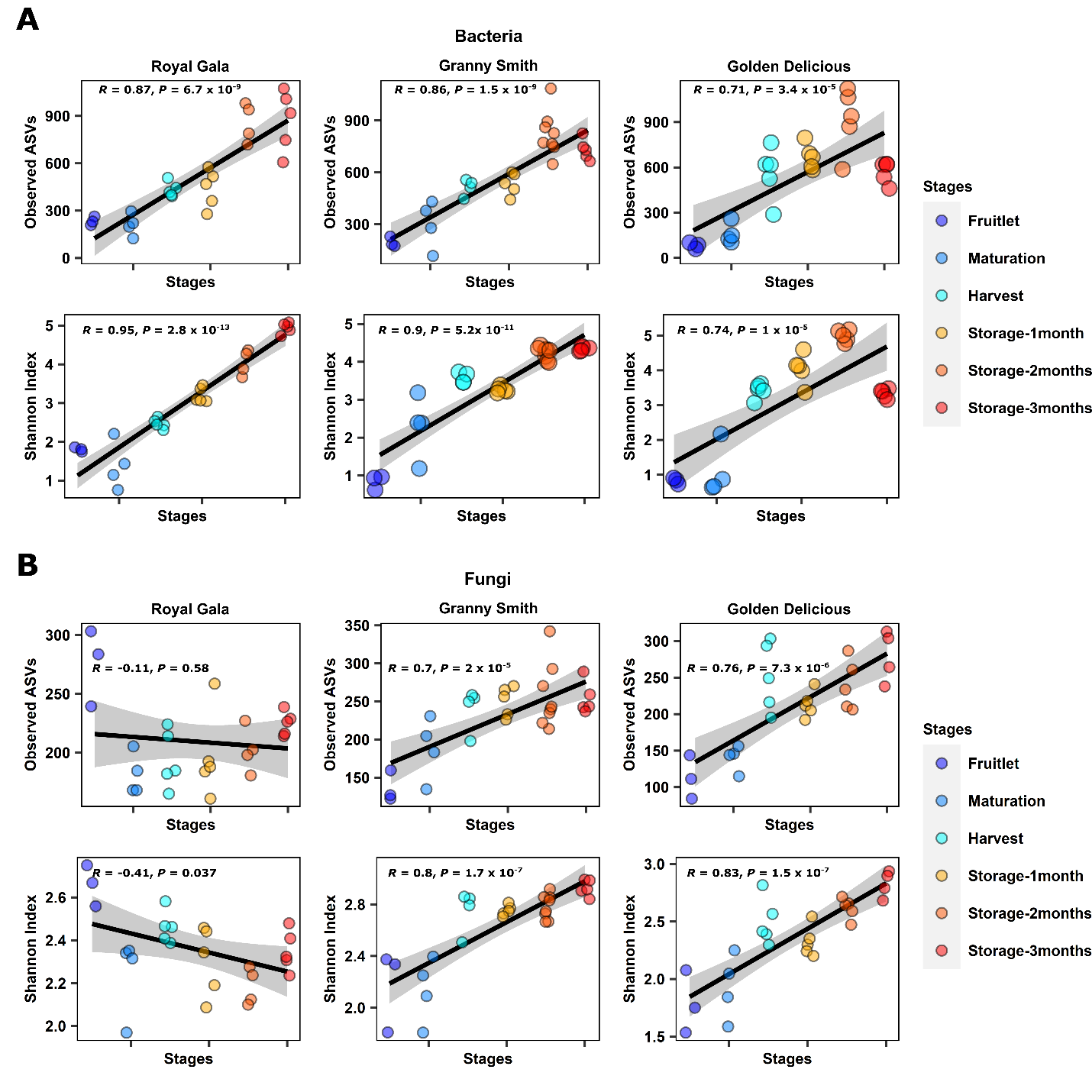


**Supplementary Figure 6: Fruit developmental stages and storage periods correlates with the carposphere microbial alpha diversity.**

Strong positive correlation (*P* < 0.0001) between the fruit developmental stages and storage periods (plotted at the x-axis) and richness (Observed ASVs) as well as within-sample diversity (Shannon Index) in the apple carposphere microbiome in both (**a**) bacteria and (**b**) fungi except for fungi in ‘Roya Gala’. Note the absence of increase or correlation in fungal community richness or diversity in ‘Royal Gala’ cultivar. The linear regression coeffecients are shown at the bottom right of each figures.


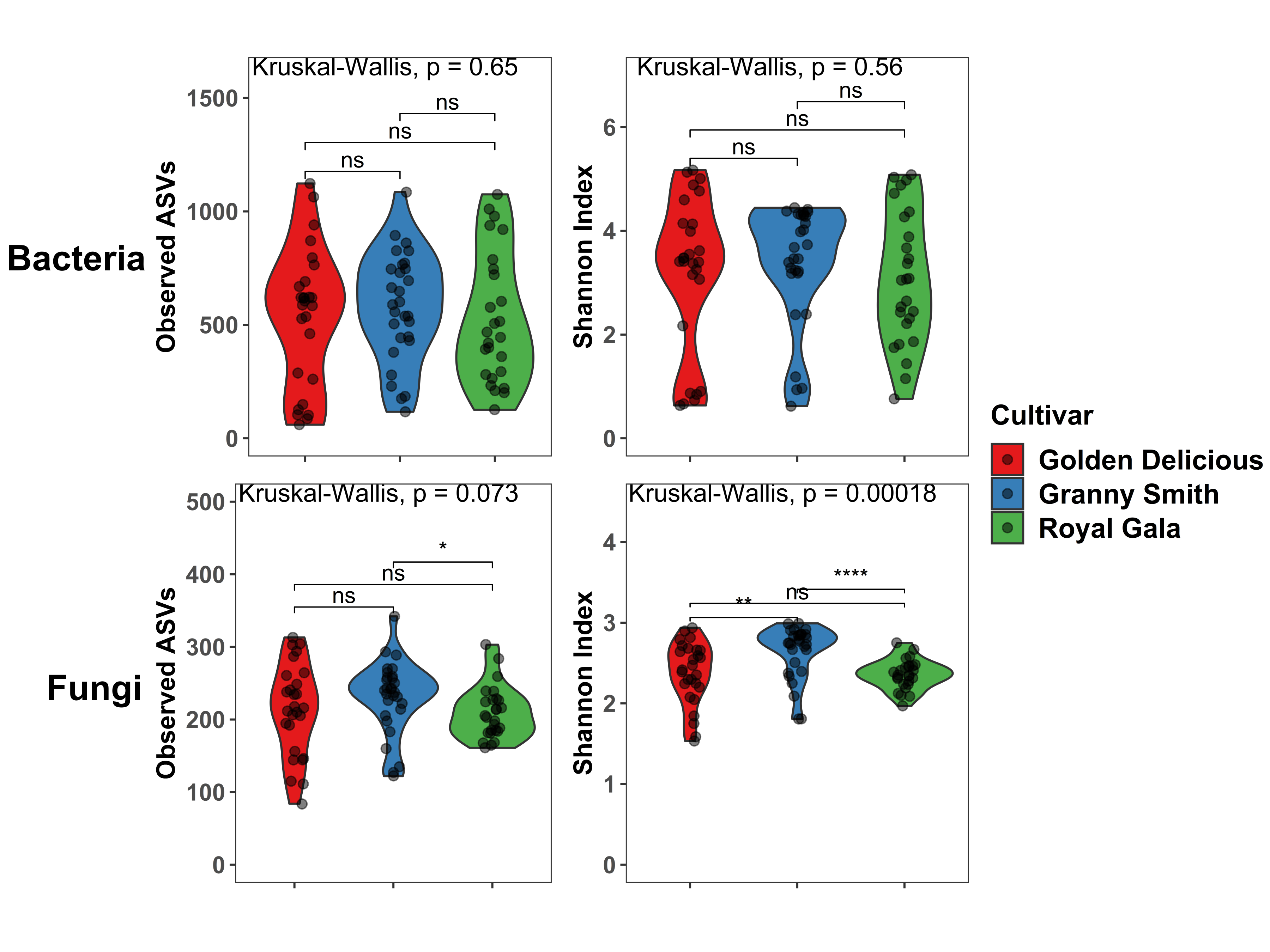


**Supplementary Figure 7: Cultivar-wise comparison of the carposphere microbial alpha diversity.**

Violin plots showing comparisons of α- diversity indices between the three cultivars. No significant difference (Kruskal-Wallis test) was observed in the bacterial community (Observed ASVs: *P* = 0.065; Shannon index: *P* < 0.056) while in case of the fungal community, a significant difference was seen in Observed ASVs (*P* = 0.00018) where ‘Royal Gala’ samples had lower Observed ASVs as well as Shannon Index.


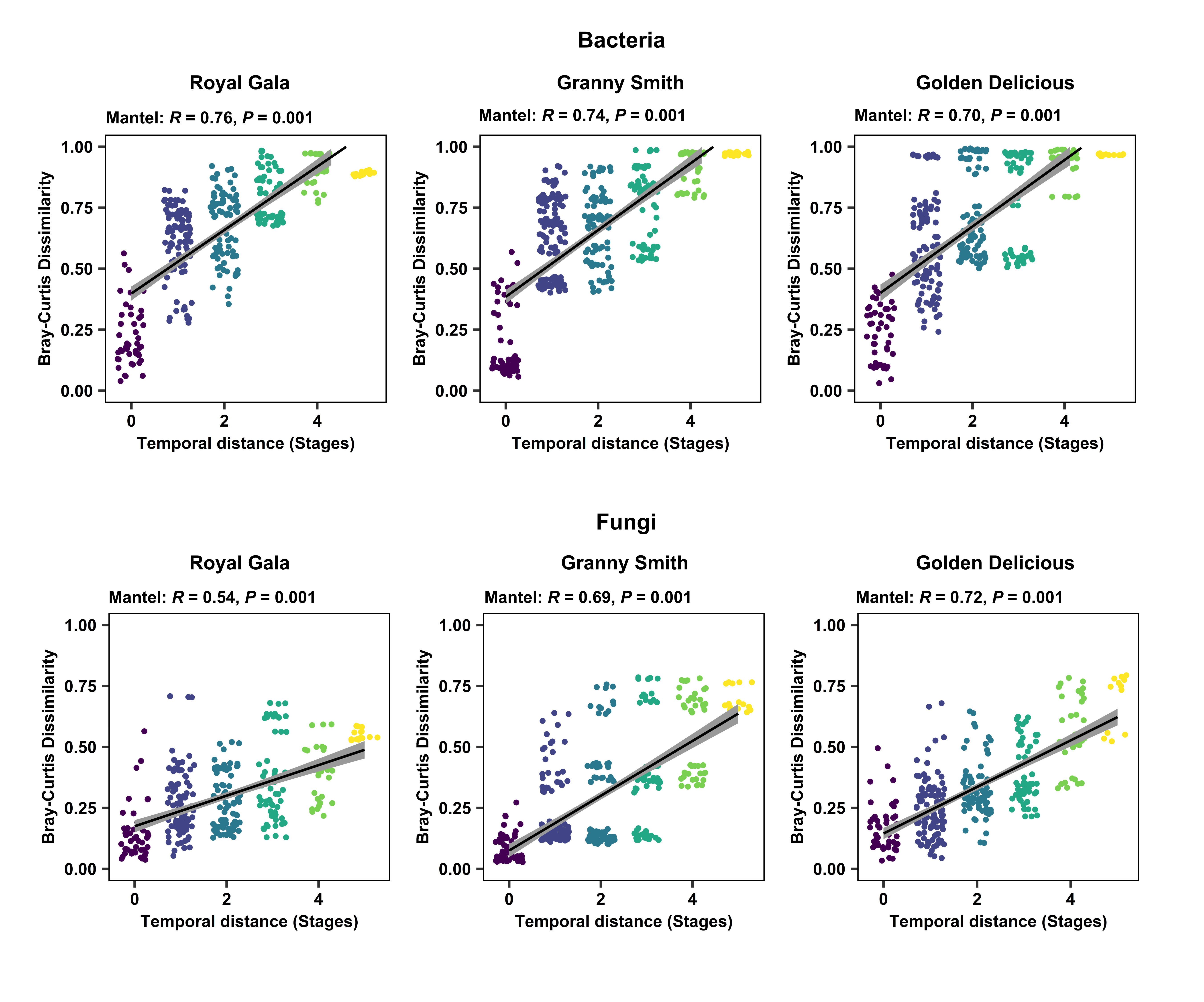


**Supplementary Figure 8: Strong community succession of the apple carposphere microbiome observed in three apple cultivars.**

Mantel correlation between temporal distance (fruit stages) and Bray–Curtis community dissimilarity showed strong succession in both bacterial and fungal communities in the three apple cultivars.

**
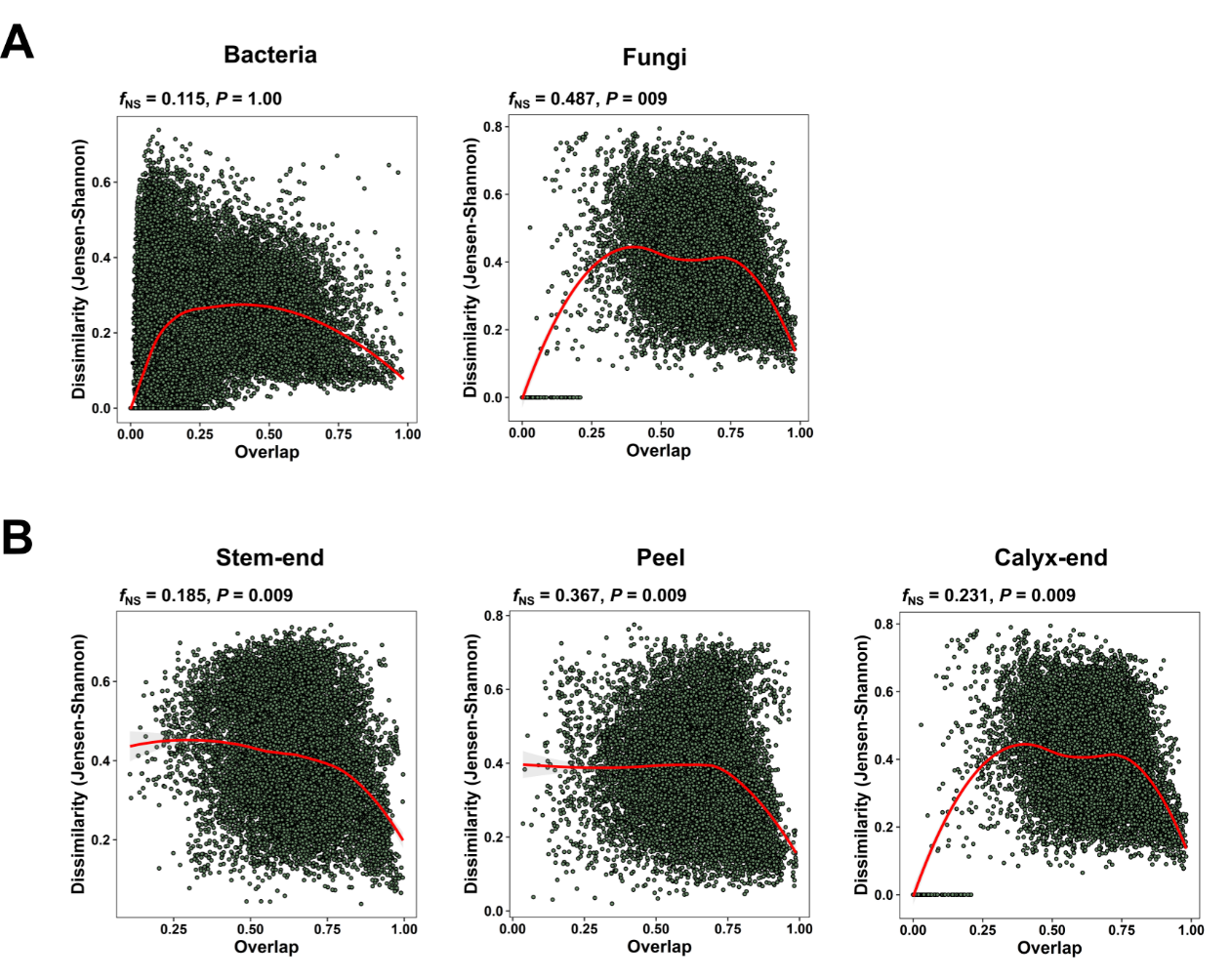
**

**Supplementary Figure 9: Universal ecological dynamics in fruit carposphere microbiomes.**

(**a**) Dissimilarity-overlap curves (DOC) for all bacterial and fungal samples of apple carposphere microbiomes from different geographical locations and tissue types showing significant negative slopes (*P* = .009) in fungi but not in bacteria. For DOCs, the overlap and dissimilarity of all the sample pairs were calculated and represented each sample pair as a point in the dissimilarity–overlap plane. (**b**) Universality was observed at varying levels when fungal microbiomes of the same data set were analyzed separately for different tissues with highest *f*_NS_ value in peel tissues, followed by calyx end and the least at the stem end.
